# Development of a Multidimensional Pain Questionnaire in Professional Dance (MPQDA): a pilot study

**DOI:** 10.1186/s13102-022-00580-5

**Published:** 2022-11-03

**Authors:** Jasmin Haenel, Thomas Schoettker-Koeniger, Eileen M. Wanke

**Affiliations:** 1 Institute of Occupational, Social and Environmental Medicine, Goethe-University Frankfurt am Main, Theodor-Stern-Kai 7, 60590 Frankfurt, Germany; 2 Faculty of Social Work and Health, HAWK-University of Applied Sciences and Arts, Goschentor 1, 31134 Hildesheim, Germany

**Keywords:** Pain, Professional dance, Questionnaire, Validity, Reliability, ROC analysis

## Abstract

**Background:**

Pain is part of the everyday life of professional dancers. It can indicate health risks and impair the ability to work. Suitable screening tools can be used to identify pain and its risk potential. A comprehensive, multidimensional, differentiated assessment tool for pain in professional dance does not currently exist.

**Methods:**

An initial questionnaire was developed in German and English and was assessed in a qualitative pretest. In a field study with a cross-sectional design including n = 72 dancers from Germany (n = 36 responses each in the English and German language versions), the questionnaire was optimized by item analysis, its psychometric properties (dimensionality, construct validity, reliability) were examined and the ability of the pain dimensions to classify the subjective ability to work in training was analyzed (ROC analysis).

**Results:**

The developed *Multidimensional Pain Questionnaire in Professional Dance* (MPQDA) was reduced and optimized in its psychometric properties. Following questions were reduced in their items or answer categories: pain localizations (from 20 to 15 regions), accompanying symptoms (from 6 to 3 items), sensory and affective pain quality (from 20 to 10 items), pain frequency (from 4 to 3 answer categories), and the motives of working with pain (from 14 to 12 items). Regarding the subjective ability to work in training, the variables of the ability to work in rehearsals and in performances, as well as the accompanying symptoms of tension and mobility restrictions, showed a relatively good classification ability (Area under the Curve (AUC) ≥ 0.7 in the 95% confidence interval) and significant, moderate to strong correlations (Somers' D > 0.25, *p* < 0.05). The classification ability of the other pain dimensions was largely absent or poor.

**Conclusion:**

The MPQDA differentiates various pain dimensions in professional dancers and is available in a compatible manner in German and English. The clinical relevance needs to be explored further in the future.

**Supplementary Information:**

The online version contains supplementary material available at 10.1186/s13102-022-00580-5.

## Background

Experiencing pain is unavoidable for dancers, especially for those who enter a professional career. Pain is considered ‘everyday’ or ‘normal’ amongst dancers [1, 2]. Various studies have revealed a high prevalence of pain, including that of Thomas and Tarr [3] who were able to identify pain associated with dancing in 78% of (pre-)professional contemporary dancers. In the study by Jacobs et al. [4], pain was found to impair dancing in 38.8% of ballet dancers and 45.1% of modern dancers. Several studies of ballet dancers in Sweden have demonstrated a very high 12-month prevalence of musculoskeletal pain of over 90% [5–7].

According to the *International Association for the Study of Pain* (IASP), pain is ‘an unpleasant sensory and emotional experience associated with, or resembling that associated with, actual or potential tissue damage’ [8]. In addition, pain is a complex construct with various dimensions. Birbaumer and Schmidt's pain model differentiates between sensory, affective, vegetative and motor components, pain assessment and pain behavior [9]. Furthermore, the temporal course, in the sense of acute and chronic pain [9], is an important dimension. Pain is understood as being a personal experience influenced by biopsychosocial factors [8]. In the course of life experience, a concept of pain is acquired [8].

In the socio-cultural context of dance, pain is not principally experienced as ‘negative’ (‘bad’ pain/injury pain), but can also be experienced as a ‘positive’ sensation (‘good’ pain/performance pain) [1–3, 10]. Pain is more or less perceived as a health and occupational threat by dancers. ‘Positive’ pain is primarily associated with achievement, improvement and an increase in pain tolerance. In contrast, ‘negative’ pain impairs dancing and can cause consequential disorders [1–3]. However, the described experience of positive pain is not a phenomenon specific to dance, but can be observed in athletes in general [11].

Due to the unavoidable confrontation with pain in dance, dancers have to find a way to deal with it. Often, however, no conscious addressing or coping emerges in pain behavior; rather, pain is often ignored and dancing continues regardless [1–3]. This behavior carries various health risks, including chronicity and the provocation of consequential injuries [2, 3]. This pain coping and pain behavior are similarly common in athletes.

Since the body is highly relevant as a working tool in dance [12], musculoskeletal pain can threaten the professional practice and, thus, the livelihood of dancers. In order to prevent health and occupational impairments, it is important to identify the risk potential of pain in professional dancers by using appropriate screening tools. According to the characteristics of different pain dimensions, such as intensity or the temporal course, pain can signalize a health risk to a more or less extent. Becoming aware of and reflecting on the characteristics of pain can be important not only for dancers but also for health care providers. Depending on the pain assessment, appropriate pain management should be applied.

Various pain measurement instruments already exist [13]; there are also specific pain assessments for dance. The *Self-Estimated Functional Inability because of Pain* (SEFIP) is a screening questionnaire developed for dance [14]. Fourteen body regions are rated on a five-point scale from ‘very well’ (= 0) to ‘cannot work in the production because of pain’ (= 4) [14]. However, the questionnaire has deficits; these include, for example, combining the dimensions of pain intensity and the ability to work in dance which should be recorded separately. The *Dance Functional Outcome Survey* (DFOS) [15] has also revealed heterogeneity in the pain dimensions in the classification of pain. The response option ‘I have occasional pain with strenuous dance or exercise. I don’t think that things are entirely back to normal. Limitations are mild and tolerable if I am careful.’ [15] contains information on temporal occurrence of pain, on pain occurrence under mechanical stimuli, on pain assessment, on functional limitations and on behavior. In addition, the DFOS focuses on functional requirements in dance [15] rather than pain as a specific construct. Lampe et al. [16] emphasize the need for the development and validation of differentiated pain assessment instruments in dance, taking into account the sociocultural circumstances. Although there are many similarities in pain experience and behavior between dancers and athletes, sociocultural circumstances are different. In professional dance exist specific work structures, such as working in training, rehearsals and performances [12]. Furthermore, there are certain social actors in the lifeworld of dancers that can influence the experience and behavior of pain. For example, choreographers can put pressure on dancers [1]. In order to address the sociological component in a biopsychosocial pain assessment tool, it is necessary to include characteristics of the lifeworld of professional dancers.

The aim of this research was to develop a Multidimensional Pain Questionnaire for Professional Dance (MPQDA). The questionnaire was developed for piloting in Germany, as the authors had good field access into the dance scene for this country. A challenge in the questionnaire's development in Germany was to create an equivalent questionnaire in both German and English, since the working language in the dance scene is often English. The questionnaire was initially developed on the basis of theory and content considerations (face validity). As differences in the understanding of the questionnaire are conceivable, depending on the language version, a qualitative pretest was completed with five dancers with different linguistic backgrounds. In a pilot study, the psychometric properties were examined and optimized on the basis of cross-sectional data. This step was carried out for the total sample as well as in comparison of the language versions. Furthermore, it was investigated as to what extent the pain dimensions of the MPQDA can classify the subjective ability to work with pain in training (dance technique classes) as a relatively permanent and standardized component in the daily work of dancers.

## Methods

**Fig. 1:**
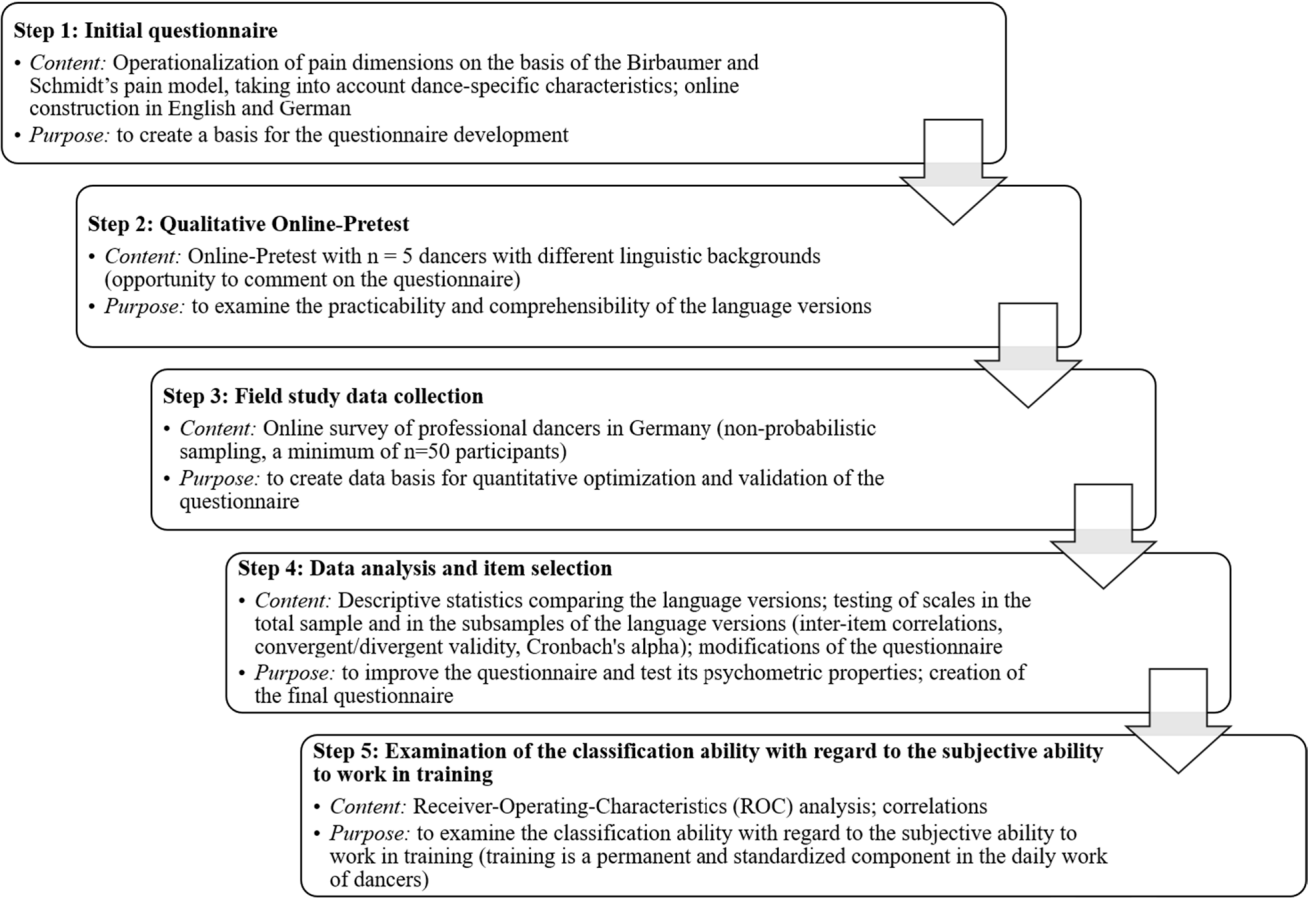
Development process of the Multidimensional Pain Questionnaire in Dance (MPQDA)

Figure 1 illustrates the development process of the questionnaire.

### Initial content of the questionnaire

The *Multidimensional Pain Questionnaire in Dance* (MPQDA) is based on the survey by Lampe et al. [17, 18] concerning pain in semi- and non-professional dance, which was then modified and adapted for professional dancers. The theoretical basis is Birbaumer and Schmidt's pain model [9]. In addition, the time course was considered with regard to acute and chronic pain [9, 19, 20]. The questionnaire includes the following blocks (questions contents are in parentheses):

- prevalence and localizations (three-month pain prevalence, pain localizations, localization of most severe pain, accompanying symptoms)
- subjective sensation of pain (pain intensity, sensory and affective pain quality)
- temporal course of pain (pain duration and frequency, sudden/creeping onset of pain, pain occurrence under mechanical stimuli)
- pain assessment (positive/negative pain)
- pain behavior (subjective work ability, motives for working with pain)

Block a) contained the three-month pain prevalence collected via a no/yes selection during or within 24 h after working time (training, rehearsals, and performances). Only participants who reported pain answered the subsequent pain questions. The period of three months was based on the survey period of the validated *Pain Sensation Scale* (SES) according to Geissner [21]. Pain localizations in the previous three months were inquired by multiple selection from six categories of the head and trunk region, six categories of the upper extremity (right and left, respectively) and eight categories of the lower extremity (right and left, respectively). The selection options were based on existing assessments such as the *Nordic Musculoskeletal Questionnaire* (NMQ) [22] or the *Self-Estimated Functional Inability because of Pain* (SEFIP) [14]. In the subsequent question, the most affected region was to be selected from these categories of localizations, to which all further pain questions referred. On a four-point ordinal scale from ‘not’ (= 0) to ‘very’ (= 3), accompanying symptoms (tension, redness, swelling, warming, restrictions of mobility and resilience) were asked.

Block b) contained the average perceived pain intensity on the 11-point *Numeric Rating Scale* (NRS) from ‘no pain’ (= 0) to ‘worst conceivable pain’ (= 10) [13]. Furthermore, pain quality was assessed by rating twelve sensory and eight affective adjectives on a four-point ordinal scale ranging from ‘does not apply’ (= 0) to ‘applies exactly’ (= 3). The scale was based on existing pain adjective lists: *McGill Pain Questionnaire* (MPQ) by Melzack [23], the *Pain Sensation Scale* (SES) by Geissner [21] and the *Pain Description List* (SBL) by Korb and Pfingsten from the *German Pain Questionnaire* (DSF, version 2015.2) [24, 25]. The adjectives were chosen from content considerations which were based on the dancers' descriptions of pain in Thomas and Tarr's study [3].

In block c), the duration of pain was surveyed using five categories ranging from a maximum of one week to longer than six months. The time periods were oriented to the classification of acute, subacute and chronic pain [9, 20]. The pain frequency in the selected period could be chosen as a subcategory from ‘only once’ (= 0) to ‘permanently’ (= 3). Furthermore, the manner of pain occurrence was asked, i.e., if it occurred as a sudden traumatic event, as a creeping process, as an occurrence of pain within 24 h after working time or ‘in another way’ (with the option of an open entry). The frequency of pain occurrence under the mechanical stimuli of weight-bearing and movement, or at rest, was asked on a four-point ordinal scale from ‘never’ (= 0) to ‘permanently’ (= 3).

In block d), an 11-point bipolar rating scale was used to record the pain assessment with regard to ‘good/positive’ pain as rather harmless and not disturbing (values from + 1 to + 5) and ‘bad/negative’ pain as alarming and disturbing (values from -1 to -5). There was the opportunity to choose the middle of the scale (= 0) if the pain could not be classified as ‘good’ or ‘bad’.

Block e) asked whether work was done in training, rehearsals or performances despite experiencing pain (subjective ability to work); response options were ‘No’ (= 2), ‘Yes, with limitations’ (= 1) and ‘Yes, without limitations’ (= 0). If work was carried out despite pain, motives for this behavior were subsequently asked. Fourteen items formulated on the basis of the known motives for dancing with pain or injury from the literature [1, 4, 26] were rated on a four-point ordinal scale from ‘does not apply’ (= 0) to ‘applies exactly’ (= 3).

Sociodemographic and health-related information were recorded as co-variables; these included gender, age, height, weight, injuries, diseases and smoking. Information on the participants' professional practice included the years of professional work as a dancer, the employment relationship (freelance/salaried), the predominant dance style (classical/neoclassical, contemporary/dance theater, musical/revue, other) and information on the average amount of training, rehearsals and performances in hours per week.

### Online construction and pretest of the questionnaire

The questionnaire was provided via the survey server SoSci Survey [27] in German and in English. The authors (German native speakers, English as a second language) translated the questionnaire from German into English and the translation was subsequently checked by an English native speaker (German as a second language).

In order to examine qualitatively the practicability and comprehensibility of the German and English versions, an online pretest was conducted [28]. The pretest was conducted from 22/05/2019 to 07/06/2019 by five dancers with different linguistic backgrounds (mother language English, German or another). Two questionnaires were completed in the German version and three in the English version. In the pretest, comments were permitted in addition to the regular response. Furthermore, additional questions (e.g., on the meaningfulness of the questions and ordering) were asked, based on recommendations available on the construction of written surveys [29–31]. Minor adjustments were then made as a result of the pretest (e.g., the word ‘currently’ was added to the question about injuries).

### Study design and population

In a pilot study, the questionnaire was field-tested and optimized using cross-sectional data. A sample size of at least n = 50 was sought, a size which is recommended as a minimum requirement for validation studies [32]. The target group included professional dancers in Germany above the age of 18 with sufficient German or English language skills. Excluded individuals were persons younger than 18 years as well as dancers from the semi-, non- and pre-professional areas. A cover letter was included which informed the participants about the study. The participants were only included if they actively agreed to the consent question on the first page. An ethical vote was obtained via the Ethics Committee of the Department of Medicine of the Goethe University Frankfurt am Main (No. 25/19).

### Data collection

The sampling was conducted by a non-probabilistic method. Access to the questionnaire was via the electronic distribution of the questionnaire's link at dancers' workplaces (e-mails to theaters), dance associations (Tanzmedizin Deutschland e. V. (ta.med), Bundesdeutsche Ballett- und Tanztheaterdirektor*innen-Konferenz (BBTK), Stiftung TANZ, Dancersconnect, Dachverband Tanz) and social media (Facebook, tanznetz.de). The survey period was from 27/02/2020 to 07/04/2020.

### Data processing

A total of n = 82 participants completely answered the questionnaire. The raw data were checked according to the inclusion and exclusion criteria, the logical sequence of the response and the plausibility. Five participants had experienced no pain in the last 3 months; these cases were eliminated from further analysis as they had consequently not answered the main questionnaire containing the in-depth questions on pain. Four cases were eliminated in which professional work as a dancer appeared questionable (n = 3 with a total weekly work time of < 10 h and n = 1 being a tournament sports trainer). Another case with > 50% missing values was also eliminated. Thus, a total sample (TS) of n = 72 cases were included in the analysis (n = 36 each in the English and German language versions).

### Data analysis

Data were analyzed using Stata/IC 14.2. Based on descriptive statistics, for the dichotomous variables, items were dropped or combined if fewer than 5 participants in the TS had selected the respective item; for the four-point ordinal scales, items were dropped if more than 70% in the TS had selected the ‘not’ (= 0) or ‘does not apply’ (= 0) category of the respective item. Descriptive statistics were then calculated for the total sample (TS), English version (EV) and German version (GV). Differences between the language versions were calculated using the Chi^2^-test, Fisher's exact test, the Mann–Whitney-U-test or the t-test for independent samples. The significance level was α = 0.05. For non-normally distributed metric variables, the median ($\widetilde{x}$) and interquartile range (IQR) were specified and non-parametric test procedures were used.

Scales with numerous items (dichotomous: pain localizations according to the subscales ‘head and torso’, ‘upper extremity’ and ‘lower extremity’; four-point ordinal scales: accompanying symptoms, sensory and affective pain quality, pain occurrence under mechanical stimuli and psychosocial motives for working with pain) were checked in several steps by statistical parameters. Items were dropped or combined if necessary. Firstly, for an examination of the dimensionality or homogeneity of the items of a scale, inter-item correlations were computed by means of tetrachoric correlation for the dichotomous variables and polychoric correlation for the four-point ordinal scales. Items were dropped if they did not correlate with any of the other items of the scale (r_pol_ or r_tet_ < 0.2). In addition, item removal was considered when the correlation was very high (r_pol_ or r_tet_ > 0.9) [32]. Secondly, in the course of convergent and divergent validity, Pearson correlations between the items of a scale and the rest sum score of the scale (without including the respective item to be tested in the sum score) was calculated [33]. The threshold value for adequate convergent validity was set at r = 0.3; this value is known from the literature as the threshold value in the course of item-total correlation [32]. If the correlation was not high enough, item removal was considered. Divergent validity is defined by the proportion of items that have a greater correlation coefficient with the sum score of their own dimension than with the score of another scale [33]. Thirdly, as a reliability measure, internal consistency was calculated using Cronbach's alpha (> 0.9 excellent, > 0.8 good, > 0.7 acceptable, > 0.6 questionable, > 0.5 poor and < 0.5 unacceptable) [32, 34]. On the basis of the different calculation procedures, which were carried out for the TS as well as for the EV and GV, and the content-related considerations, the authors decided whether a sum score formation (summation of the individual item values of a scale) of the respective scales would be appropriate.

The sum scores or the individual items were included in the further analysis. Since most of the pain dimensions did not show significant differences between the EV and the GV, the authors consider the language versions to be comparable in terms of content. Therefore, to address the following research question, the TS was used for further analysis.

*Receiver-Operating-Characteristics* (ROC) analysis was used to examine the extent to which the pain dimensions of the MPQDA can classify the subjective ability to work in training. For this purpose, the variable of subjective work ability in training was dichotomized into no work ability (‘No’) or limited work ability (‘Yes, with limitations’) (= 1) and full work ability (‘Yes, without limitations’) (= 0). Nominal variables of pain dimensions were each coded as dichotomous dummy variables. Furthermore, the items of the psychosocial motives for working with pain were recoded into ‘does not apply’ (= 3) to ‘applies exactly’ (= 0), since a full ability to work (= 0) is more likely with the stronger manifestation of the motives. Subsequently, correlations (point-biserial correlation, Somers' D, Cramer's V) of the pain dimensions, as well as the sociodemographic, health and occupational data, with the subjective work ability in training (dichotomous) were calculated. Next, in the course of the ROC analysis of the pain dimensions, the *Area under the Curve* (AUC), as a measure of discriminability [32, 35] of the single pain dimensions, was determined with a 95% confidence interval. Discriminability was assumed if the AUC was > 0.5 [35]. An AUC of ≥ 0.7 was considered acceptable [32]. Moreover, for metric and ordinal variables, it was judged whether the ROC curve was a proper or improper curve. An improper curve was judged as being present when the curve crossed the 0.5 change diagonal [35].

## Results

### Study population

Table 1 shows the sociodemographic, health and occupational data of the study population. Dancers who chose the GV had a significantly higher age (*p* < 0.001), years of professional work (*p* < 0.001), percentages of disease (*p* = 0.003) and freelance work (*p* < 0.001) than those who responded in the EV. The EV sample showed a significantly higher average time spent working in rehearsals per week (*p* < 0.001). The samples of the EV (M = classical/neoclassical) and the GV (M = contemporary/dance theater) were also significantly different in the predominantly practiced dance styles (*p* < 0.001).

**Table 1:** Sociodemographic, health and occupational data of the dancers in the total sample (n = 72) and in the language versions (n = 36 each)

	Total (n = 72)	English (n = 36)	German (n = 36)	*p* value
Gender n (%)				0.29^a^
Female	52 (72.2)	24 (66.7)	28 (77.8)	
Male	20 (27.8)	12 (33.3)	8 (22.2)	
Age (years)*				0.00^d^
$\overline{x }$ (sd)	30.3 (8.6)	26.0 (4.6)	34.5 (9.7)	
Height (cm)				0.80^d^
$\overline{x }$ (sd)	167.8 (7.9)	167.6 (7.7)	168.0 (8.2)	
Weight (kg)				0.25^d^
$\overline{x }$ (sd)	57.9 (9.5)	56.6 (9.1)	59.2 (9.9)	
Body mass index (kg/m^2^)				0.097^d^
$\overline{x }$ (sd)	20.5 (2.1)	20.0 (1.8)	20.9 (2.3)	
Injuries n (%)				0.15^a^
No	44 (61.1)	25 (69.4)	19 (52.8)	
Yes	28 (38.9)	11 (30.6)	17 (47.2)	
Diseases* n (%)				0.003^a^
No	50 (69.4)	31 (86.1)	19 (52.8)	
Yes	21 (29.2)	5 (19.9)	16 (44.4)	
Missing [n (%)]	[1 (1.4)]	–	[1 (2.8)]	
Smoking n (%)				0.22^b^
No	50 (69.4)	23 (63.9)	27 (75.0)	
Yes, ≤ 10 cigarettes/day	18 (25.0)	12 (33.3)	6 (16.7)	
Yes, > 10 cigarettes/day	4 (5.6)	1 (2.8)	3 (8.3)	
Years of professional work*				0.004^d^
$\overline{x }$ (sd)	9.1 (7.7)	6.5 (4.5)	11.7 (9.3)	
Missing [n (%)]	[1 (1.4)]	[1 (2.8)]	–	
Employment relationship* n (%)				0.00^a^
Freelance	29 (40.3)	5 (13.9)	24 (66.7)	
Salaried	43 (59.7)	31 (86.1)	12 (33.3)	
Dance style* n (%)				0.002^b^
Classical/neoclassical	26 (36.1)	19 (52.8)	7 (19.4)	
Contemporary/dance theater	35 (48.6)	16 (44.4)	19 (52.8)	
Musical/revue	2 (2.8)	0	2 (5.6)	
Other	9 (12.5)	1 (2.8)	8 (22.2)	
Working hours (per week) $\widetilde{x}$ (IQR), Missing [n (%)]				
Training	7.5 (2.5)	7.5 (1.0)	7.75 (6.0)	0.85^c^
Rehearsals*	20.0 (20.0) [2 (2.8)]	29.5 (10.0) –	20.0 (12.0) [2 (5.6)]	0.00^c^
Performances	3.0 (2.5) [9 (12.5)]	3.0 (2.5) [1 (2.8)]	2.5 (3.5) [8 (22.2)]	0.60^c^

$\widetilde{x}$ = median, IQR = interquartile range, $\overline{x }$ = mean, sd = standard deviation

^a^Chi^2^-test

^b^Fisher's exact test

^c^Mann–Whitney U test

^d^t-test

*p < 0.05

### Descriptive statistics: modifications and comparison of the language versions

#### Localizations

For the ‘head and torso’ regions, ‘chest/thorax’ (n = 2, 2.8%) was eliminated because less than five subjects of the TS had selected it. For the upper extremity regions, the ‘upper arm’ (right: n = 1, 1.4%; left: n = 2, 2.8%), the ‘forearm’ (right: n = 2, 2.8%; left: n = 1, 1.4%) and the ‘hand’ (right: n = 4, 5.6%; left: n = 3, 4.2%) were rarely selected; these were combined with the neighboring regions: ‘shoulder/upper arm’, ‘elbow/forearm’ and ‘hand/wrist’. For the lower extremity, ‘forefoot and toes (except big toe)’ (right only: n = 3, 4.2%) was rarely selected, therefore, this was combined with the neighboring region ‘big toe’ to yield the ‘forefoot’ (this was also applied for the left side). The selection options for the question about the most affected pain region were also changed accordingly. The accompanying symptoms of the most affected pain region of ‘reddened’, ‘swollen’ and ‘warm’ were eliminated because more than 70% in the TS had selected these as ‘not’ (= 0).

A summary of the frequencies of pain regions and of the most severely affected pain region in the previous 3 months in the TS, EV and GV is provided in the Additional file 1: Tables A1 and A2. The right forefoot was significantly more often affected in the GV (n = 11, 30.6%) than in the EV (n = 4; 11.1%; *p* = 0.04). In the remaining categories, there were no significant differences. There were also no significant differences between the EV and the GV in the accompanying symptoms "tight/hard/tense" and "restricted in mobility" (Additional file 1: Table A3). However, resilience was found to be ‘fairly’ limited in the GV ($\widetilde{x}$ = 2.0, IQR = 0) and ‘somewhat’ limited in the EV ($\widetilde{x}$ = 1.0, IQR = 1.5) (*p* < 0.001).

#### Subjective sensation of pain

In this block, modifications were applied to the pain adjectives: For sensory pain adjectives, the terms ‘throbbing’, ‘tingling’, ‘burning’ and ‘searing’ were dropped and, for the affective adjectives, ‘ghastly’ and ‘dreadful’ were eliminated because more than 70% of the TS had indicated them as ‘does not apply’ (= 0).

Pain intensity on the NRS was statistically comparable in the EV ($\widetilde{x}$ = 6.0, IQR = 2.0) and the GV ($\widetilde{x}$ = 5.0, IQR = 4.0; *p* = 0.36). The median and IQR values of the sensory and affective adjectives of the TS, EV and GV are shown in the Additional file 1: Table A4). Significant differences between the language versions were found for the pain sensations "shooting" (EV: $\widetilde{x}$ = 0, IQR = 0; GV: $\widetilde{x}$ = 1.0, IQR = 3.0; *p* = 0.002) and "fearful" (EV: $\widetilde{x}$ = 0, IQR = 1.0; GV: $\widetilde{x}$ = 0.5, IQR = 2.0; *p* = 0.046).

#### Temporal course of pain

In the block of the temporal course of pain, the original answer ‘only once’ for the pain frequency was deleted since no one had selected this option.

The statistics of pain duration and frequency, manner of pain occurrence and pain occurrence under mechanical stimuli are shown in the Additional file 1: Table A5. Pain occurrence when weight-bearing was significantly more pronounced in the GV ($\widetilde{x}$= 2.0; 25%-percentile = 2; 75%-percentile = 3) than in the EV ($\widetilde{x}$= 2.0; 25%-percentile = 1; 75%-percentile = 2) (*p * = 0.01). In other variables of the temporal course of pain, there were no significant differences.

#### Pain assessment

Dancers in the EV and in the GV rated pain as being rather ‘bad/negative’ with $\widetilde{x}$ (IQR) = − 2.0 (2.0) (*p* = 0.07).

#### Pain behavior

Table 2 shows the frequencies of subjective ability to work in training, rehearsals and performances. There were no significant differences.

**Table 2:** Frequencies of the subjective ability to work in training, rehearsals and performances of the dancers in the total sample (n = 72) and in the language versions (n = 36 each)

Do you work despite pain?	Total (n = 72) n (%)	English (n = 36) n (%)	German (n = 36) n (%)	*p* value
In training				0.79^a^
No	1 (1.4)	1 (2.8)	0	
Yes, with limitations	53 (73.6)	27 (75.0)	26 (72.2)	
Yes, without limitations	18 (25.0)	8 (22.2)	10 (27.8)	
In rehearsals				1.00^a^
No	3 (4.2)	1 (2.8)	2 (5.6)	
Yes, with limitations	48 (66.7)	24 (66.7)	24 (66.7)	
Yes, without limitations	21 (29.2)	11 (30.6)	10 (27.8)	
In performances				0.37^a^
No	7 (9.7)	2 (5.6)	5 (13.9)	
Yes, with limitations	24 (33.3)	11 (30.6)	13 (36.1)	
Yes, without limitations	41 (56.9)	23 (63.9)	18 (50.0)	

^a^Fisher's exact test

A total of n = 71 dancers worked in training, rehearsals and/or performances despite pain. The psychosocial motives for this behavior are presented in the Additional file 1: Table A6. There were significant differences in the items ‘I feel existential/financial pressure.’ (*p* = 0.0001), ‘I don't want to lose my status.’ (*p* = 0.02) and ‘I have concerns my dance skills are going down.’ (*p* = 0.02); these were more pronounced in the GV than in the EV. The item ‘The pain’s not so bad, so there’s no need for a break.’ was significantly more predominant in the EV than in the GV (*p* = 0.04).

### Item analysis, validity and reliability of scales

The correlation matrices for the inter-item correlations in the TS, EV and GV are shown in the Additional file 2. Table 3 shows the Cronbach's alpha values and the convergent and divergent validities of the scales with numerous items.

**Table 3:** Cronbach's alpha values and the convergent and divergent validities of the scales in the total sample (n = 72) and in the language versions (n = 36 each)

Dimension	Total (n = 72)			English (n = 36)			German (n = 36)		
	α	CV	DV	α	CV	DV	α	CV	DV
Head and torso (5 body regions)	0.54	9/25 36.6%	20/25 80.0%	0.40	9/25 36.6%	12/25 48.0%	0.63	17/25 68.0%	17/25 68.0%
Upper extremity (3 body regions right/left)	0.65			0.58			0.74		
Lower extremity (7 body regions right/left)	0.53			0.49			0.55		
Accompanying symptoms (3 items)	0.49	1/3 33.3%	–	0.70	3/3 100%	–	0.21	0/3 0.0%	–
Sensory pain quality (5 items)	0.73	10/10 100%	8/10 80.0%	0.73	7/10 70.0%	9/10 90.0%	0.73	9/10 90.0%	8/10 80.0%
Affective pain quality (5 items)	0.81			0.70			0.86		
Pain occurrence when weight-bearing/during movement (2 items)	0.31	1/3 33.3%	3/3 100%	0.26	1/3 33.3%	3/3 100%	0.46	1/3 33.3%	3/3 100%
Pain occurrence at rest (1 item)	–			–			–		
Motives of working with pain (12 items)	0.89	12/12 100%	–	0.92	12/12 100%	–	0.84	10/12 83.3%	–

α = Cronbach's alpha; CV = convergent validity (absolute and relative proportion of items having r > 0.3 with the sum score of their own dimension); DV = divergent validity (absolute and relative proportion of items having a higher correlation coefficient with the sum score of their own dimension than with the score of another dimension)

#### Localizations

The tetrachoric correlation matrices for the head and torso, upper extremity and lower extremity regions showed partly perfect correlations (r_tet_ = 1.0). However, we decided not to perform an item deletion since the large number of selectable regions may have caused individual cases to have these perfect correlations by coincidence. The correlations were often very small (r_tet_ < 0.2). However, there was no item that correlated very low with all other items. The statistical parameters had a low quality, with the exception of the upper extremity (Table 3). For the content considerations, reasons of practicability and simply for sufficient internal consistency, a sum score was generated for the head and torso, upper extremity and lower extremity regions.

#### Accompanying symptoms

The polychoric correlation matrix did not show very high correlations of r_pol_ > 0.9. Only in the GV did the item of tension (‘tight/hard/tense’) correlate modestly with the other two items on the restrictions of mobility and of resilience (r_pol_ < 0.2). Since the statistical parameters in the TS and also in the GV were insufficient (Table 3), a sum score was not useful, thus, the items were treated as single items.

#### Sensory and affective pain quality

The polychoric correlation matrices of the remaining eight sensory and six affective adjectives did not show very high correlations of r_pol_ > 0.9. Even though correlations were small in parts, there was no item that correlated as being very small with r_pol_ < 0.2 with all other items of the scale. After an initial analysis, the terms ‘dull’, ‘pressing’ and ‘cramping’ were dropped for the sensory adjectives because they showed limited sufficient convergent and divergent validities (with the exception of the ‘cramping’ item in the GV). For affective pain quality, the item ‘tiring/exhausting’ was removed since it did not show sufficient convergent validity in the TS and also the EV and GV (r < 0.3). The statistical parameters of the sensory pain scale, consisting of five items (pulling, tearing, shooting, stabbing, sharp), and the affective scale, consisting of five items (fearful, wretched, terrible, paralyzing, unbearable), were judged to be good (Table 3), thus, sum scores could be formed.

#### Pain occurrence under mechanical stimuli

In the polychoric correlation matrix, the item of pain occurrence at rest correlated negatively with the other two items in the TS and both the EV and GV; it was, therefore, separated. The statistical parameters were found to be insufficient (Table 3) and, thus, it was not possible to form a sum score.

#### Psychosocial motives for working with pain

In the polychoric correlation matrix, only two coefficients in the EV were at r_pol_ > 0.9. The item ‘The pain’s not so bad, so there’s no need for a break.’ did not correlate with any other item in the TS (r_pol_ < 0.2). However, in the EV and GV, this item showed slightly higher correlations with some other items. After analysis of the scale, the items ‘The pain’s not so bad, so there’s no need for a break.’ and ‘The pain is a natural consequence of dancing.’ were eliminated because they did not show sufficient convergent validity in the TS as well as in the EV and GV (r < 0.3). The scale of the psychosocial motives for working with pain, consisting of 12 items, showed very good statistical parameters (Table 3) and, thus, it was possible to form a sum score.

### Classifying ability with regard to the subjective ability to work in training

The sociodemographic data (gender, age, height, weight, body mass index), health-related data (injuries, diseases, smoking) and occupational data (years of professional work, employment relationship, dance style, working hours) did not correlate significant with the subjective ability to work in training (correlation coefficients < 0.2, *p* < 0.05).

Most of the pain dimension variables correlated very weakly and non-significantly with the subjective ability to work in training (Table 4). Classifying ability was absent (AUC < 0.5) or very low for most variables (Table 4). An AUC ≥ 0.7 was included at the 95%-CI only for the variables of tension, mobility and resilience limitations, pain intensity, sensory pain quality and subjective work ability in rehearsals and in performances. However, intensity, sensory pain quality and work ability in rehearsals showed improper curves. Among the seven variables with an AUC ≥ 0.7 in the 95%-CI, the variables of tension, mobility restriction and work ability in rehearsals and in performances showed significant, positive correlations, ranging from moderate to strong, with the subjective ability to work in training.

**Table 4:** Correlations and ROC analysis of the pain dimensions with regard to the dancers' subjective ability to work in training (dichotomous) (n = 72)

	Correlations		ROC analysis		Missing n (%)
	Correlation coefficient	*p* value	AUC (95%-CI)	ROC-Curve	
Localizations (sum scores)					
Head and torso	r_pbis_ = 0.04	0.74	0.54 (0.38–0.698)	IPC	–
Upper extremity	r_pbis_ = 0.13	0.27	0.55 (0.42–0.68)	PC	–
Lower extremity	r_pbis_ = − 0.08	0.50	0.47 (0.31–0.64)	IPC	–
Localization of most severe pain (dichotomous)					
Head and torso	V = 0.03	0.78	0.52 (0.38–0.65)	–	–
Upper extremity	V = 0.17	0.14	0.56 (0.51–0.598)	–	–
Lower extremity	V = 0.13	0.28	0.43 (0.29–0.56)	–	–
Accompanying symptoms					
Tension*	D = 0.29	0.03	0.65 (0.51–0.78)	PC	–
Mobility restriction*	D = 0.39	0.01	0.695 (0.55–0.84)	PC	–
Resilience restriction	D = 0.12	0.41	0.56 (0.41–0.71)	PC	–
Pain intensity (NRS)	D = 0.12	0.44	0.56 (0.41–0.70)	IPC	4 (5.6)
Pain quality (sum scores)					
Sensory	r_pbis_ = 0.06	0.599	0.57 (0.396–0.75)	IPC	–
Affective	r_pbis_ = − 0.01	0.91	0.49 (0.33–0.65)	IPC	–
Pain duration	D = − 0.10	0.50	0.45 (0.28–0.61)	IPC	2 (2.8)
Pain frequency	D = − 0.21	0.12	0.39 (0.26–0.53)	PC	2 (2.8)
Pain occurrence (dichotomous)					
Sudden/single event	V = 0.22	0.06	0.58 (0.53–0.64)	–	1 (1.4)
Over time/creeping*	V = 0.37	0.002	0.29 (0.19–0.39)	–	1 (1.4)
Within 24 h after work	V = 0.18	0.14	0.58 (0.499–0.65)	–	1 (1.4)
In another way	V = 0.12	0.29	0.55 (0.47–0.62)	–	1 (1.4)
Pain occurrence					
When weight-bearing	D = − 0.13	0.37	0.43 (0.29–0.58)	PC	4 (5.6)
During movement*	D = − 0.34	0.03	0.33 (0.18–0.48)	PC	5 (6.9)
At rest	D = − 0.05	0.78	0.48 (0.31–0.65)	IPC	3 (4.2)
Pain assessment	D = − 0.14	0.37	0.43 (0.28–0.58)	IPC	1 (1.4)
Subjective work ability					
In rehearsals*	D = 0.61	0.00	0.80 (0.67–0.94)	IPC	–
In performances*	D = 0.41	0.00	0.70 (0.59–0.81)	PC	–
Psychosocial motives for working with pain (sum scores)	r_pbis_ = 0.01	0.92	0.52 (0.36–0.67)	IPC	5 (6.9)

r_pbis_ = Point-biserial correlation coefficient, D = Somers' D, V = Cramer's V, *p < 0.05;

ROC = Receiver-Operating-Characteristics, AUC = Area under the Curve, 95%-CI = 95%-Confidence Interval, PC = Proper Curve, IPC = Improper Curve

## Discussion

The *Multidimensional Pain Questionnaire in Professional Dance* (MPQDA) is, to our knowledge, the first pain questionnaire that seeks to capture diverse pain dimensions in a differentiated manner for professional dance. At this stage of development, the questionnaire initially created (face validity) was optimized, in the context of a field study, by means of item analysis, its psychometric properties regarding dimensionality, construct validity (convergent/divergent validity) and reliability (internal consistency) which were all tested; the classifying ability of the pain dimensions regarding subjective ability to work in training was also analyzed.

### Language versions

In a variety of sociodemographic, health and occupational data, the dancers of the EV differed significantly from those of the GV (Table 1). However, in the descriptive statistics of the pain questions, only a few items showed significant differences between the language versions. Thus, the items "shooting" and "fearful" differed significantly in the samples of the language versions. The translations of "shooting" and "fearful" in the MPQDA correspond to the translations of these adjectives from studies on the validation of German translations of the English McGill questionnaire [36, 37]. Therefore, the authors do not assume a translation problem, but rather a random difference in pain perception in the EV and GV samples.

Furthermore, in the item analysis as well as in the validity and reliability examination of scales with several items, the results were largely compatible in the language versions. It can, therefore, be assumed that the language versions of the MPQDA are compatible.

### Item analysis, validity and reliability of the scales

The item analysis made it possible to shorten the questionnaire and to optimize the psychometric properties of the scales. Nevertheless, the generation of a sum score could not be recommended for all scales constructed from the initial considerations; this concerned the items of accompanying symptoms (tension, restriction of mobility and of resilience) and items of pain occurrence (when weight-bearing, during movement or at rest). That tension and the occurrence of pain at rest are to be separated from the movement- and load-associated factors still seems logical. However, why the latter two factors could not each be combined into one dimension remains questionable. The study by Wanke et al. [38] used the preliminary version of the questionnaire in a survey of pain in dance teachers, which differed from the initial version of the MPQDA mainly in the information on professional practice and in the motives of working with pain. In addition, the questionnaire was only available in German. In the cited study [38] the sum score ‘Functional impairment’ could be formed from the items of mobility and resilience restrictions while the sum score ‘Biomechanical exposure’ could be formed from the items of pain occurrence during movement and when weight-bearing, as the statistical parameters were found to be sufficient. Both the present study and that of Wanke et al. [38] used non-probabilistic methods in the sampling. However, the sample in Wanke et al. [38] was double the size of this study at n = 143 compared to n = 72, which may make the cited study more representative. Furthermore, it is conceivable that the use of the questionnaire in the target group of dance teachers is not comparable to its use in professional dancers.

### Classifying ability with regard to the subjective ability to work in training

The sociodemographic, health and occupational data collected were not associated with the subjective work ability in training and appear to be less relevant in this regard. However, this conclusion should be made with caution to the extent that, for example, the study by Jacobs et al. [4] identified the years of dancing professionally as a determinant of occupation-limiting pain (SEFIP ≥ 3).

In addition, a large proportion of the pain dimensions of the MPQDA appear to be less relevant, as the correlation coefficients and the AUC were low in many cases (Table 4). It should be considered that the variable to be classified was a pain behavior from the subjective perspective of dancers. In the everyday work of dancers, pain is often accepted and ignored [1–3]. Anderson and Hanrahan [10] found that the dancers' cognitive appraisals and coping strategies for pain did not differ in terms of the pain experience as performance or injury pain or from the pain intensity. Thus, dancers seem to adapt their behavior less to the type of pain they experience. This may have resulted in most of the pain dimensions of the MPQDA being only weakly associated with the subjective work ability in training and, thus, poorly able to classify work ability in that regard. Therefore, it can be concluded that a combination of pain dimensions (e.g., pain intensity with pain behavior in dance), as is the case in the SEFIP [14], is not useful in surveying pain in professional dancers. The developed MPQDA differentiates the pain dimensions in the survey; this should be emphasized as a strength when compared to other existing assessments in dance.

It should be taken into account that the answers concerning the subjective ability to work "No" and "Yes, with limitations" were combined for the ROC analysis. It is obvious that there were very few dancers who are unable to work in relation to those who work with limitations. It is possible that the number of those who are unable to work is underrepresented. However, it is conceivable that due to the prevalent behavior of ignoring pain and continuing to dance [1–3], the numbers may be quite representative. If a larger number of dancers who do not work were available and these were compared with those who dance with or without limitations, a change in the correlation measures and the discriminatory ability of the MPQDA is conceivable.

Furthermore, it is conceivable that health care providers (e.g., physicians, physical therapists) would make divergent behavioral recommendations regarding the ability of affected dancers to work, based on the pain dimensions surveyed in the MPQDA. Discrepancies between the recommendations of health care providers and the behaviors of dancers are known. According to Lai et al. [39], dancers tend to be unwilling to change their behavior in the long term and do not accept breaks in training. Therefore, based on the health care providers' assessments of work ability using the MPQDA, which presumably differ from the dancers' subjective judgments, a change in the correlation measures and discriminatory ability of the MPQDA is likely.

There also emerged, however, variables that showed relatively good classifiability with regard to the subjective work ability in training (AUC ≥ 0.7 in the 95%-CI) and correlated moderately to strongly. If a dancer had limited work ability in training, he or she was also more likely to have limited work ability in rehearsals and in performances. Furthermore, for the accompanying symptoms, the dimensions of tension and mobility and resilience restrictions showed relatively good classifiability and positive correlations; the more severe these were, the more likely was a limited ability to work in training. Ramel and Moritz [6] also identified muscular tension before performances as a risk factor for pain associated with limitations in the dancers' ability to work. Wanke et al. [38] identified ‘Functional impairment’ (sum score of mobility and resilience restrictions) as a determinant of pain intensity in dance teachers. In addition, for pain intensity and sensory pain quality, the 95%-CI of the AUC included the value 0.7, with both variables showing an improper curve in the ROC analysis as well as being weakly and non-significantly correlated with the subjective work ability in training. A proper classifying ability of these pain dimensions does not seem to have been established.

### Application

The MPQDA was developed for use in professional dancers. It contains specific information on professional practice and is tailored to the work structure and sociocultural environment of professional dancers (subjective work ability, motives for working with pain). For screening and monitoring of pain in practice, the pain questions can be used separately, i.e., irrespective of the survey of sociodemographic, health-related, and occupational questions. For practical use by health care providers, the questionnaire does not represent a conclusive pain assessment. If, for example, functional impairments or the occurrence of pain under weight-bearing or during movement are reported, it should be further investigated with which dance elements (e.g., plié or lifting a partner) these complaints are associated.

The blocks a) prevalence and localizations, b) subjective sensation of pain, c) temporal course of pain and d) pain assessment may also be applied to other athletes. However, the application in a different population has to be validated. Pain data from other athletes could thus be compared with those from the dance population.

### Limitations and recommendations for future research

One limitation of this study is that the translation of the questionnaire did not involve a multi-step process of translation and cultural adaptation as recommended in the literature [40, 41]. However, it can be assumed that the language versions are sufficiently comparable: Firstly, the questionnaire was translated forward and pretested using a small sample of participants with different language backgrounds. Secondly, there were hardly any differences between the language versions in the pain dimensions among the population of professional dancers in Germany.

Furthermore, pain is a highly complex, multifactorial phenomenon and the MPQDA does not claim to be entirely comprehensive. Thus, for example, pain behavior was captured as the subjective ability to work. However, pain behavior can also include, for instance, medical/therapeutic treatment.

A limitation of the pilot study is the small sample size of n = 72. In the GV and EV subsamples, each with n = 36, the recommended minimum sample size of n = 50 for validation studies [32] was not met. A reason why a larger number of dancers could not be reached could have been the beginning restrictions in the cultural sector in Germany in the Corona pandemic in March 2020 [42]. However, since conclusions in the course of item analysis, validity and reliability of scales with several items were made on the basis of the calculations in the TS, EV and GV, the calculations and decisions for item removal or combination should be sufficiently valid.

Furthermore, due to the small sample size, certain statistical methods for determining dimensionality, such as factor analyses, were not applicable as these methods require minimum sample sizes of n = 100 [32]. The dimensionality was determined on the basis of correlations. For further validation of the MPQDA, larger samples should be investigated in order to explore the MPQDA with regard to its multidimensionality and the interactions of the pain dimensions using methods such as factor analyses or structural equation models.

In addition, it is important to evaluate further the MPQDA in terms of its clinical relevance in future studies. Hence, the classifying ability of pain dimensions in relation to the judgment of work ability by health care providers should also be evaluated. Furthermore, in the course of cross-sectional data collection of this first examination of the MPQDA, it is unclear as to what extent the questionnaire can predict (consequential) injuries or the chronicity of the complaints and where the thresholds lie in the risk classification of pain in dancers. In the future, this should be verified within longitudinal studies and, thus, the MPQDA should be further developed as a screening tool for professional dancers. In additionally, other properties of the questionnaire, such as test–retest reliability, need to be examined.

## Conclusion and prospects

The *Multidimensional Pain Questionnaire in Professional Dance* (MPQDA), which was developed in this study, differentiates the various pain dimensions in professional dancers and is available in a compatible manner in both the German and English languages (availability of the questionnaires with information on scoring can be found in the Additional files 3 and 4). Even though the correlations and the classifying abilities regarding the subjective work ability with pain in training of most of the pain dimensions of the MPQDA were not, or scarcely, established, the clinical relevance of the dimensions has not been conclusively clarified. In this regard, the MPQDA needs to be further examined.

## Supplementary Information

**Additional file 1**. Descriptive statistics of pain dimensions comparing the language versions.

**Additional file 2**. Inter-item correlations of scales with numerous items.

**Additional file 3**. Multidimensional Pain Questionnaire in Professional Dance (MPQDA). [English version].

**Additional file 4**. Multidimensionaler Schmerzfragebogen im professionellen Tanz (MPQDA). [German version].

## Data Availability

The datasets used and analysed during this study are available from the corresponding author on reasonable request.

## Abbreviations

AUC: Area under the Curve
DFOS: Dance Functional Outcome Survey
EV: English version
GV: German version
MPQ: McGill Pain Questionnaire
MPQDA: Multidimensional Pain Questionnaire in Professional Dance
NMQ: Nordic Musculoskeletal Questionnaire
NRS: Numeric Rating Scale
ROC: Receiver-Operating-Characteristics
SEFIP: Self-Estimated Functional Inability because of Pain
SES: Pain Sensation Scale
SBL: Pain Description List (from the German pain questionnaire)
TS: Total sample
