# Supplementary material for: Development of a Multidimensional Pain Questionnaire in Professional Dance (MPQDA): a pilot study
Source: BMC Sports Sci Med Rehabil. 2022 Nov 3;14:189. doi: 10.1186/s13102-022-00580-5 (PMC9635190; doi:10.1186/s13102-022-00580-5)
Supplement: Supplementary file 1 — Additional file 1. Descriptive statistics of pain dimensions comparing the language versions. [file 13102_2022_580_MOESM1_ESM.pdf]

Additional file 1:
Descriptive statistics of pain dimensions comparing the language versions

Content

1 Localizations (Tables A1-A3) 1
2 Subjective sensation of pain (Table A4) 2
3 Temporal course of pain (Table A5) 3
4 Pain behavior (Table A6) 4

1 Localizations (Tables A1-A3)

Table A1. Pain localizations in the last 3 months of the dancers in the total sample (n = 72) and in the language versions (n = 36 each)

	**Total (n = 72)** n (%)		**English (n =36)** n (%)		**German (n = 36)** n (%)		**p-value**	
**Head and torso**								
Head	7 (9.7)		1 (2.8)		6 (16.7)		0.12[b]	
Neck /cervical spine	39 (54.2)		20 (55.6)		19 (52.8)		0.81[a]	
Upper back/ thoracic spine	24 (33.3)		14 (38.9)		10 (27.8)		0.32[a]	
Lower back/ lumbar spine/ iliosacral joint	47 (65.3)		24 (66.7)		23 (63.9)		0.80[a]	
Stomach	5 (6.94)		2 (5.6)		3 (8.3)		1.00[b]	
**Upper extremity**	**right**	**left**	**right**	**left**	**right**	**left**	**right**	**left**
Shoulder/ upper arm	13 (18.1)	13 (18.1)	8 (22.2)	8 (22.2)	5 (13.9)	5 (13.9)	0.36[a]	0.36[a]
Elbow/ forearm	7 (9.7)	5 (6.9)	5 (13.9)	2 (5.6)	2 (5.6)	3 (8.3)	0.43[b]	1.00[b]
Wrist/ hand	13 (18.1)	14 (19.4)	7 (19.4)	7 (19.4)	6 (16.7)	7 (19.4)	0.76[a]	1.00[a]
**Lower extremity**	**right**	**left**	**right**	**left**	**right**	**left**	**right**	**left**
Hip joint	17 (23.6)	26 (36.1)	10 (27.8)	15 (41.7)	7 (19.4)	11 (30.6)	0.41[a]	0.33[a]
Upper thigh	9 (12.5)	8 (11.1)	7 (19.4)	6 (16.7)	2 (5.6)	2 (5.6)	0.15[b]	0.26[b]
Knee	26 (36.1)	28 (38.9)	13 (36.1)	14 (38.9)	13 (36.1)	14 (38.9)	1.00[a]	1.00[a]
Lower leg	6 (8.3)	12 (16.7)	5 (13.9)	8 (22.2)	1 (2.8)	4 (11.1)	0.199[b]	0.21[a]
Ankle joint	16 (22.2)	22 (30.6)	9 (25.0)	12 (33.3)	7 (19.4)	10 (27.8)	0.57[a]	0.61[a]
Rear-/midfoot	9 (12.5)	8 (11.1)	4 (11.1)	3 (8.3)	5 (13.9)	5 (13.9)	1.00[b]	0.71[b]
Forefoot (right*)	15 (20.8)	17 (23.6)	4 (11.1)	6 (16.7)	11 (30.6)	11 (30.6)	0.04[a]	0.17[a]

[a]Chi^2-test, [b]Fisher's exact test, *$p<0.05$

Table A2. Most severely affected pain region of the dancers in the total sample (n = 72) and in the language versions (n = 36 each) according to superior categories of pain regions

	**Total**<br>**(n = 72)**<br>n (%)	**English**<br>**(n =36)**<br>n (%)	**German**<br>**(n = 36)**<br>n (%)	**p-value**
Head and torso	30 (41.7)	13 (36.1)	17 (47.2)	
Upper extremity	6 (8.3)	3 (8.3)	3 (8.3)	
Lower extremity	36 (50.0)	20 (55.6)	16 (44.4)	0.64[b]

[b]Fisher's exact test

Table A3. Accompanying symptoms of the most severely affected pain region (not = 0, somewhat = 1, fairly = 2, very = 3) of the dancers in the total sample (n = 72) and in the language versions (n = 36 each)

	**Total**<br>**(n = 72)**<br>$\tilde{x}$ (IQR)	**English**<br>**(n =36)**<br>$\tilde{x}$ (IQR)	**German**<br>**(n = 36)**<br>$\tilde{x}$ (IQR)	**p-value**
Tight/hard/tense	2.0 (2.0)	2.0 (2.0)	2.0 (2.0)	0.92[c]
Restricted in mobility	2.0 (1.0)	2.0 (1.0)	2.0 (1.0)	0.61[c]
Less resilient*	2.0 (1.0)	1.0 (1.5)	2.0 (0)	0.00[c]

[c]Mann-Whitney-U-test; *p<0.05

2 Subjective sensation of pain (Table A4)

Table A4. Sensory and affective pain quality (does not apply = 0, applies a little = 1, applies mostly = 2, applies exactly = 3) of the dancers in the total sample (n = 72) and in the language versions (n = 36 each)

	**Total**<br>**(n = 72)**<br>$\tilde{x}$ (IQR)<br>Missing [n (%)]	**English**<br>**(n = 36)**<br>$\tilde{x}$ (IQR)<br>Missing [n (%)]	**German**<br>**(n = 36)**<br>$\tilde{x}$ (IQR)<br>Missing [n (%)]	**p-value**
**Sensory pain quality**				
Dull	0 (1.0)<br>[4 (5.6)]	0 (1.0)<br>[2 (5.6)]	0.5 (1.0)<br>[2 (5.6)]	0.38[c]
Pressing	1.0 (2.0)<br>[5 (6.9)]	1.0 (1.0)<br>[3 (8.3)]	1.0 (2.0)<br>[2 (5.6)]	0.55[c]
Cramping	0.5 (1.0)<br>[4 (5.6)]	0 (1.0)<br>[3 (8.3)]	1.0 (2.0)<br>[1 (2.8)]	0.28[c]
Pulling	1.0 (2.0)<br>[2 (2.8)]	1.0 (2.0)<br>[2 (5.6)]	2.0 (1.5)<br>-	0.69[c]
Tearing	0 (2.0)<br>[4 (5.6)]	1.0 (2.0)<br>[3 (8.3)]	0 (2.0)<br>[1 (2.8)]	0.48[c]
Shooting*	0 (1.0)<br>[4 (5.6)]	0 (0)<br>[3 (8.3)]	1.0 (3.0)<br>[1 (2.8)]	0.002[c]
Stabbing	0 (2.0)<br>[6 (8.3)]	0 (2.0)<br>[4 (11.1)]	1.0 (2.0)<br>[2 (5.6)]	0.34[c]
Sharp	0 (2.0)<br>[2 (2.8)]	1.0 (2.0)<br>[1 (2.8)]	0 (2.0)<br>[1 (2.8)]	0.93[c]

Affective pain quality				
Tiring/exhausting	1.0 (2.0)<br>[2 (2.8)]	1.0 (2.0)<br>[2 (5.6)]	1.0 (2.0)<br>-	0.67^{c}
Fearful*	0 (1.0)<br>[4 (5.6)]	0 (1.0)<br>[2 (5.6)]	0.5 (2.0)<br>[2 (5.6)]	0.046^{c}
Wretched	0 (1.0)<br>[6 (8.3)]	0 (0)<br>[3 (8.3)]	0 (1.0)<br>[3 (8.3)]	0.12^{c}
Terrible	0 (1.0)<br>[5 (6.9)]	0 (1.0)<br>[3 (8.3)]	0 (1.0)<br>[2 (5.6)]	0.67^{c}
Paralyzing	0 (1.0)<br>[5 (6.9)]	0 (1.0)<br>[3 (8.3)]	0 (1.0)<br>[2 (5.6)]	0.49^{c}
Unbearable	0 (1.0)<br>[4 (5.6)]	0 (1.0)<br>[2 (5.6)]	0 (1.0)<br>[2 (5.6)]	0.99^{c}

$\tilde{x}$ = median, IQR = interquartile range, ^{c}Mann-Whitney-U-test; *p<0.05

3 Temporal course of pain (Table A5)

Table A5. Pain duration, pain frequency (only once = 0, rarely = 1, frequently = 2, permanently = 3), type of pain occurrence, pain occurrence under mechanical stimuli (rarely = 1, frequently = 2, permanently = 3) of the dancers in the total sample (n = 72) and in the language versions (n = 36 each)

	**Total (n = 72)**	**English (n = 36)**	**German (n = 36)**	**p-value**
**Pain duration**<br>n (%)				
Maximum of one week	9 (12.5)	6 (16.7)	3 (8.3)	0.86^{b}
Between one and 6 weeks	11 (15.3)	6 (16.7)	5 (13.9)	
Between 6 and 12 weeks	8 (11.1)	4 (11.1)	4 (11.1)	
Between 3 and 6 months	13 (18.1)	7 (19.4)	6 (17.7)	
Longer than 6 months	29 (40.3)	13 (36.1)	16 (44.4)	
Missing [n (%)]	[2 (2.8)]	-	[2 (5.6)]	
**Pain frequency**				
$\tilde{x}$ (IQR)	2.0 (0)	2.0 (1.0)	2.0 (0)	0.27^{c}
Missing [n (%)]	[2 (2.8)]	-	[2 (5.6)]	
**Type of pain occurrence**<br>n (%)				
Suddenly/ a single event	9 (12.5)	6 (16.7)	3 (8.3)	0.48^{b}
Over time/ creeping	41 (56.9)	19 (52.8)	22 (61.1)	
Within 24 hours after work	12 (16.7)	7 (19.4)	5 (13.9)	
In another way	9 (12.5)	3 (8.3)	6 (16.7)	
Missing [n (%)]	[1 (1.4)]	[1 (2.8)]	-	

**Pain occurrence under mechanical stimuli** $\tilde{x}$ (IQR), Missing [n (%)]				
When weight-bearing*	2.0 (0) [4 (5.6)]	2.0 (1.0) [2 (5.6)]	2.0 (1.0) [2 (5.6)]	0.01[c]
During movement	2.0 (1.0) [5 (6.9)]	2.0 (1.0) [1 (2.8)]	2.0 (0) [4 (11.1)]	0.46[c]
At rest	1.0 (1.0) [3 (4.2)]	1.0 (1.0) [3 (8.3)]	1.0 (1.0) -	0.72[c]

$\tilde{x}$ = median, IQR = interquartile range; [b]Fisher's exact test, [c]Mann-Whitney-U-test; *p<0.05

4 Pain behavior (Table A6)

Table A6. Psychosocial motives of working with pain (does not apply = 0, applies a little = 1, applies mostly = 2, applies exactly = 3) of the dancers in the total sample (n = 71) and in the language versions (English: n = 35, German: n = 36)

	**Total (n = 71)** $\tilde{x}$ (IQR) Missing [n (%)]	**English (n = 35)** $\tilde{x}$ (IQR) Missing [n (%)]	**German (n = 36)** $\tilde{x}$ (IQR) Missing [n (%)]	**p-value**
The pain's not so bad, so there's no need for a break.*	1.0 (1.0) [2 (2.8)]	2.0 (1.0) [1 (2.9)]	1.0 (2.0) [1 (2.9)]	0.04[c]
The pain is a natural consequence of dancing.	1.0 (1.0) [1 (1.4)]	1.0 (1.0) -	1.0 (2.0) [1 (2.8)]	0.78[c]
I don't want to let my company down.	2.0 (2.0) [2 (2.8)]	2.0 (2.0) -	1.0 (3.0) [2 (5.6)]	0.97[c]
I feel existential/ financial pressure.*	1.0 (2.0) [2 (2.8)]	0 (1.0) [1 (2.9)]	2.0 (3.0) [1 (2.8)]	0.0001[c]
I feel pressure from superiors (training leader, choreographer).	0 (2.0) [2 (2.8)]	1.0 (1.0) -	0 (2.0) [2 (5.6)]	0.51[c]
I feel pressure from colleagues.	0 (1.0) [3 (4.2)]	0 (1.0) [1 (2.9)]	0 (1.0) [2 (5.6)]	0.55[c]
I don't want to be considered unreliable.	1 (3.0) [2 (2.8)]	2.0 (2.0) -	1.0 (3.0) [2 (5.6)]	0.61[c]
I don't want to lose my role.	1.0 (2.0) [3 (4.2)]	1.0 (2.0) [1 (2.9)]	1.5 (3.0) [2 (5.6)]	0.49[c]
I don't want to lose my status.*	1.0 (2.0) [2 (2.8)]	0 (2.0) [1 (2.9)]	2.0 (3.0) [1 (2.8)]	0.02[c]
I have concerns my dancing skills are going down.*	1.0 (2.0) [2 (2.8)]	0 (2.0) [1 (2.9)]	1.0 (2.0) [1 (2.8)]	0.02[c]
I have concerns my body is getting out of its aesthetic form.	1.0 (2.0) [2 (2.8)]	0 (1.0) [1 (2.9)]	1.0 (2.0) [1 (2.8)]	0.19[c]
I want to impress the audience.	0 (1.0) [3 (4.2)]	0 (1.0) [1 (2.9)]	0 (1.0) [2 (5.6)]	0.31[c]

Dancing is more important to me than my health.	0 (1.0) [3 (4.2)]	0 (1.0) [1 (2.9)]	1.0 (1.0) [2 (5.6)]	0.11[c]
Dancing is my passion – I just have to keep dancing.	1.0 (2.5) [3 (4.2)]	1 (2.0) [1 (2.9)]	2.0 (2.0) [2 (5.6)]	0.16[c]

$\tilde{x}$ = median, IQR = interquartile range, [c]Mann-Whitney-U-test; *p<0.05
