# Supplementary material for: Development of a Multidimensional Pain Questionnaire in Professional Dance (MPQDA): a pilot study
Source: BMC Sports Sci Med Rehabil. 2022 Nov 3;14:189. doi: 10.1186/s13102-022-00580-5 (PMC9635190; doi:10.1186/s13102-022-00580-5)
Supplement: Supplementary file 2 — Additional file 2. Inter-item correlations of scales with numerous items. [file 13102_2022_580_MOESM2_ESM.pdf]

Additional file 2:
Inter-item correlations of scales with numerous items

- Calculations
  - tetrachoric correlation for the dichotomous variables (r_{tet})
  - polychoric correlation for the four-point ordinal scales (r_{pol})
- Critical values:
  - no correlation: r_{pol} or $r_{tet} < 0.2$
  - very high correlation: **r_{pol} or $r_{tet} > 0.9$**

Content

1 Localizations 3
1.1 Total sample (n = 72) 3
1.2. English version (n = 36) 4
1.3 German version (n = 36) 5
2 Accompanying symptoms 6
2.1 Total sample (n = 72) 6
2.2 English version (n = 36) 6
2.3 German version (n = 36) 7
3 Sensory and affective pain quality 7
3.1 Total sample (n = 72) 7
1.2 English version (n = 36) 8
1.3 German version (n = 36) 9

4 Pain occurrence under mechanical stimuli 9
1.1 Total sample (n = 72) 9
1.2 English version (n = 36) 10
1.3 German version (n = 36) 10
5 Psychosocial motives for working with pain 10
1.1 Total sample (n = 72) 11
1.2 English version (n = 36) 11
1.3 German version (n = 36) 12

1 Localizations

Tetrachoric correlation was performed for the following dimensions:

- **Head and torso:** head (PL04_01), neck /cervical spine (PL04_02), upper back/ thoracic spine (PL04_03), lower back/ lumbar spine/ iliosacral joint (PL04_05), stomach (PL04_06)
- **Upper extremity:** shoulder/ upper arm (right: PL05z_01; left: PL06z_01), elbow/ forearm (right: PL05z_02; left: PL06z_02), wrist/ hand (right: PL05z_03; left: PL06z_03)
- **Lower extremity:** hip joint (right: PL07_01; left: PL08_01), upper thigh (right: PL07_02; left: PL08_02), knee (right: PL07_03; left: PL08_03), lower leg (right: PL07_04; left: PL08_04), ankle joint (right: PL07_05; left: PL08_05), rear-/midfoot (right: PL07_06; left: PL08_06), forefoot (right: PL07z_07; left: PL08z_07)

1.1 Total sample (n = 72)

Head and torso:

	PL04_01	PL04_02	PL04_03	PL04_05	PL04_06
PL04_01	1.0000				
PL04_02	**1.0000**	1.0000			
PL04_03	0.3407	0.2875	1.0000		
PL04_05	**1.0000**	0.4157	0.0346	1.0000	
PL04_06	0.2490	**1.0000**	0.5915	0.2241	1.0000

Upper extremity:

	PL05z_01	PL05z_02	PL05z_03	PL06z_01	PL06z_02	PL06z_03
PL05z_01	1.0000					
PL05z_02	0.4234	1.0000				
PL05z_03	0.1246	0.4234	1.0000			
PL06z_01	0.4434	0.7523	0.2945	1.0000		
PL06z_02	0.0375	0.7731	0.5878	0.7798	1.0000	
PL06z_03	0.0876	0.1712	0.5363	0.5363	0.5626	1.0000

Lower extremity:

	PL07_01	PL07_02	PL07_03	PL07_04	PL07_05	PL07_06	PL07z_07	PL08_01	PL08_02	PL08_03	PL08_04	PL08_05	PL08_06	PL08z_07
PL07_01	1.0000													
PL07_02	0.6509	1.0000												
PL07_03	0.4322	0.2979	1.0000											
PL07_04	0.1592	0.4088	-0.3108	1.0000										
PL07_05	0.0333	-0.2565	0.1489	-0.1076	1.0000									
PL07_06	-0.0277	-1.0000	0.1317	-1.0000	0.3855	1.0000								
PL07z_07	0.3477	0.2379	0.5361	-1.0000	0.2505	0.2379	1.0000							
PL08_01	0.5311	0.2979	0.4321	-0.0407	-0.0981	-0.0455	0.1985	1.0000						
PL08_02	0.4226	0.8096	0.2086	0.4511	-0.2134	0.0000	0.2967	0.3843	1.0000					
PL08_03	-0.1928	-0.2781	0.5298	0.1534	0.2108	0.2541	0.0210	-0.1079	-0.4499	1.0000				
PL08_04	0.0298	0.3389	-0.0499	0.8512	0.2290	-0.1472	0.2627	0.0973	0.5700	0.1883	1.0000			
PL08_05	0.1016	0.0475	0.0059	0.2766	0.4877	0.2258	-0.2294	0.2122	0.2986	-0.2673	0.3398	1.0000		
PL08_06	-0.2372	-1.0000	0.2086	-1.0000	0.4497	0.6797	0.2967	-0.1817	0.0417	-0.0213	0.3936	0.6252	1.0000	
PL08z_07	-0.0020	0.1784	-0.1394	-1.0000	-0.2929	-0.0277	0.2151	-0.2655	0.0261	-0.3154	-0.1592	0.1016	0.4226	1.0000

1.2. English version (n = 36)

Head and torso:

	PL04_01	PL04_02	PL04_03	PL04_05	PL04_06
PL04_01	1.0000				
PL04_02	**1.0000**	1.0000			
PL04_03	**1.0000**	0.0408	1.0000		
PL04_05	**1.0000**	0.4899	-0.0661	1.0000	
PL04_06	-1.0000	**1.0000**	0.1398	**1.0000**	1.0000

Upper extremity:

	PL05z_01	PL05z_02	PL05z_03	PL06z_01	PL06z_02	PL06z_03
PL05z_01	1.0000					
PL05z_02	0.3284	1.0000				
PL05z_03	-0.2018	0.3927	1.0000			
PL06z_01	0.3447	0.6153	0.1437	1.0000		
PL06z_02	-1.0000	0.5391	**1.0000**	**1.0000**	1.0000	
PL06z_03	-0.2018	0.0126	0.7152	0.4237	0.4275	1.0000

Lower extremity:

	PL07_01	PL07_02	PL07_03	PL07_04	PL07_05	PL07_06	PL07z_07	PL08_01	PL08_02	PL08_03	PL08_04	PL08_05	PL08_06	PL08z_07
PL07_01	1.0000													
PL07_02	0.7270	1.0000												
PL07_03	0.2951	0.5935	1.0000											
PL07_04	0.2140	0.3927	-0.2871	1.0000										
PL07_05	-0.1338	-0.2577	0.1711	-1.0000	1.0000									
PL07_06	-1.0000	-1.0000	-0.1817	-1.0000	0.3964	1.0000								
PL07z_07	0.6631	0.7854	0.5518	-1.0000	0.0000	-1.0000	1.0000							
PL08_01	0.7207	0.5072	0.4661	-0.0269	-0.1697	0.1235	0.4801	1.0000						
PL08_02	0.6334	**0.9267**	0.2373	0.4639	-1.0000	-1.0000	0.1770	0.4086	1.0000					
PL08_03	-0.1946	-0.1945	0.5298	0.0181	0.1127	0.1656	0.1656	-0.1549	-1.0000	1.0000				
PL08_04	0.2062	0.4237	-0.2280	0.8302	-0.0000	0.0534	0.4497	0.1571	0.5123	0.2108	1.0000			
PL08_05	0.1487	-0.0941	0.1343	0.1121	0.4421	0.2529	-1.0000	0.3771	0.2877	-0.3346	0.0843	1.0000		
PL08_06	-1.0000	-1.0000	-0.0407	-1.0000	0.1343	0.8231	-1.0000	-0.1184	-1.0000	-0.0801	0.1850	0.4482	1.0000	
PL08z_07	0.1072	0.2981	-0.0499	-1.0000	-0.1864	-1.0000	0.1770	-0.1446	-0.0000	0.1883	-1.0000	-0.3294	0.3010	1.0000

1.3 German version (n = 36)

Head and torso:

	PL04_01	PL04_02	PL04_03	PL04_05	PL04_06
PL04_01	1.0000				
PL04_02	**1.0000**	1.0000			
PL04_03	0.3941	0.5548	1.0000		
PL04_05	**1.0000**	0.3394	0.1369	1.0000	
PL04_06	0.3010	**1.0000**	**1.0000**	0.0407	1.0000

Upper extremity:

	PL05z_01	PL05z_02	PL05z_03	PL06z_01	PL06z_02	PL06z_03
PL05z_01	1.0000					
PL05z_02	0.5391	1.0000				
PL05z_03	0.4639	0.4802	1.0000			
PL06z_01	0.5449	**1.0000**	0.4639	1.0000		
PL06z_02	0.3701	**1.0000**	0.3010	0.7617	1.0000	
PL06z_03	0.3927	0.4275	0.2981	0.6717	0.6575	1.0000

Lower extremity:

	PL07_01	PL07_02	PL07_03	PL07_04	PL07_05	PL07_06	PL07z_07	PL08_01	PL08_02	PL08_03	PL08_04	PL08_05	PL08_06	PL08z_07
PL07_01	1.0000													
PL07_02	0.4275	1.0000												
PL07_03	0.5935	-1.0000	1.0000											
PL07_04	-1.0000	-1.0000	-1.0000	1.0000										
PL07_05	0.2164	-1.0000	0.1248	1.0000	1.0000									
PL07_06	0.3927	-1.0000	0.3733	-1.0000	0.3927	1.0000								
PL07z_07	0.2350	-1.0000	0.5786	-1.0000	0.4764	0.4646	1.0000							
PL08_01	0.2350	-1.0000	0.4049	-1.0000	-0.0403	-0.2004	0.1415	1.0000						
PL08_02	-1.0000	-1.0000	0.1762	-1.0000	0.4275	0.5391	1.0000	0.2520	1.0000					
PL08_03	-0.1945	-1.0000	0.5298	1.0000	0.3218	0.3293	-0.0576	-0.0576	0.1398	1.0000				
PL08_04	-1.0000	-1.0000	0.2086	1.0000	0.5071	-1.0000	0.2986	-0.0959	0.6068	0.1656	1.0000			
PL08_05	0.0167	0.2922	-0.1369	1.0000	0.5313	0.2140	-0.0132	-0.0132	0.2922	-0.1946	0.6631	1.0000		
PL08_06	0.0126	-1.0000	0.3733	-1.0000	0.6717	0.5449	0.4646	-0.2004	0.5391	0.0181	0.6405	0.7565	1.0000	
PL08z_07	-0.0403	0.2520	-0.2088	-1.0000	-0.3563	0.1618	0.1415	-0.3186	0.2520	-0.6854	0.2986	0.4231	0.4646	1.0000

2 Accompanying symptoms

Polychoric correlation was performed for the following variables: tight/hard/tense (PL11_01), restricted in mobility (PL11_06), less resilient (PL11_07).

2.1 Total sample (n = 72)

	PL11_01	PL11_06	PL11_07
PL11_01	1		
PL11_06	.31493096	1	
PL11_07	.09858342	.44153582	1

2.2 English version (n = 36)

	PL11_01	PL11_06	PL11_07
PL11_01	1		
PL11_06	.57263731	1	
PL11_07	.44249671	.53508439	1

2.3 German version (n = 36)

	PL11_01	PL11_06	PL11_07
PL11_01	1		
PL11_06	.11625309	1	
PL11_07	-.15864275	.37172777	1

3 Sensory and affective pain quality

Polychoric correlation was performed for the following dimensions:

- **Sensory pain quality:** dull (SE02_03), pressing (SE02_04), cramping (SE02_05), pulling (SE02_06), tearing (SE02_07), shooting (SE02_08), stabbing (SE02_09), sharp (SE02_10)
- **Affective pain quality:** tiring/exhausting (SE02_13), fearful (SE02_14), wretched (SE02_15), terrible (SE02_18), paralyzing (SE02_19), unbearable (SE02_20)

3.1 Total sample (n = 72)

Sensory pain quality:

	SE02_03	SE02_04	SE02_05	SE02_06	SE02_07	SE02_08	SE02_09	SE02_10
SE02_03	1							
SE02_04	.39721129	1						
SE02_05	-.22833722	.12819916	1					
SE02_06	-.01191341	.14896606	.37959936	1				
SE02_07	-.19535985	.22368318	.28663394	.64744586	1			
SE02_08	-.04383884	.1481543	.17852529	.31302199	.40456077	1		
SE02_09	-.06288599	.00822011	.0113473	.32397299	.1184248	.68599824	1	
SE02_10	-.19556701	-.06940389	-.01821251	.15260294	.26056955	.71789387	.8149663	1

Affective pain quality:

	SE02_13	SE02_14	SE02_15	SE02_18	SE02_19	SE02_20
SE02_13	1					
SE02_14	.2263713	1				
SE02_15	.16421014	.54558711	1			
SE02_18	.11529735	.65233224	.66834814	1		
SE02_19	.370732	.47291957	.40477022	.74974848	1	
SE02_20	.29273063	.40359218	.3853943	.75786081	.7121409	1

1.2 English version (n = 36)

Sensory pain quality:

	SE02_03	SE02_04	SE02_05	SE02_06	SE02_07	SE02_08	SE02_09	SE02_10
SE02_03	1							
SE02_04	.28644216	1						
SE02_05	-.24692254	.12267308	1					
SE02_06	-.13676197	.06420411	.20112475	1				
SE02_07	-.22852447	.01151164	.19153514	.62604973	1			
SE02_08	.17282721	.47349418	-.18716116	.42898337	.34009543	1		
SE02_09	-.0375637	.05315171	-.22521828	.5138558	.09679106	.69455314	1	
SE02_10	-.01154903	.17955821	-.28748267	.35167368	.12973197	.68987922	.77354849	1

Affective pain quality:

	SE02_13	SE02_14	SE02_15	SE02_18	SE02_19	SE02_20
SE02_13	1					
SE02_14	.25703401	1				
SE02_15	-.1940914	-.16390123	1			
SE02_18	-.10590915	.43631164	.13244091	1		
SE02_19	.52061817	.3633298	.03292463	.7758881	1	
SE02_20	.34407775	.49381408	.21086803	**.90197653**	.89443323	1

1.3 German version (n = 36)

Sensory pain quality:

	SE02_03	SE02_04	SE02_05	SE02_06	SE02_07	SE02_08	SE02_09	SE02_10
SE02_03	1							
SE02_04	.47517612	1						
SE02_05	-.27492079	.12168418	1					
SE02_06	.10635224	.24401997	.52652628	1				
SE02_07	-.14884421	.42809328	.39736932	.72579895	1			
SE02_08	-.29894327	-.07565623	.30206223	.26741838	.57129668	1		
SE02_09	-.15168049	-.06115616	.17768293	.12698942	.19355004	.71208073	1	
SE02_10	-.37936981	-.2369683	.17272186	-.01123323	.37068109	.831637	.86829624	1

Affective pain quality:

	SE02_13	SE02_14	SE02_15	SE02_18	SE02_19	SE02_20
SE02_13	1					
SE02_14	.22600524	1				
SE02_15	.3667592	.80109277	1			
SE02_18	.24345326	.79984513	.89845917	1		
SE02_19	.26760616	.63798583	.69862272	.78790314	1	
SE02_20	.25530664	.41632854	.53360598	.65260039	.51798794	1

4 Pain occurrence under mechanical stimuli

Polychoric correlation was performed for the following variables: when weight-bearing (ZV04_01), during movement (ZV04_02), at rest (ZV02_03)

1.1 Total sample (n = 72)

	ZV04_01	ZV04_02	ZV04_03
ZV04_01	1		
ZV04_02	.22534002	1	
ZV04_03	-.07913807	-.21201348	1

1.2 English version (n = 36)

	ZV04_01	ZV04_02	ZV04_03
ZV04_01	1		
ZV04_02	.19186008	1	
ZV04_03	-.06141853	-.31101543	1

1.3 German version (n = 36)

	ZV04_01	ZV04_02	ZV04_03
ZV04_01	1		
ZV04_02	.38084932	1	
ZV04_03	-.08245727	-.08286131	1

5 Psychosocial motives for working with pain

Polychoric correlation was performed for the following variables:

- The pain's not so bad, so there's no need for a break. (SV03_01)
- The pain is a natural consequence of dancing. (SV03_02)
- I don't want to let my company down. (SV03_03)
- I feel existential/ financial pressure. (SV03_04)
- I feel pressure from superiors (training leader, choreographer). (SV03_05)
- I feel pressure from colleagues. (SV03_06)
- I don't want to be considered unreliable. (SV03_07)
- I don't want to lose my role. (SV03_08)
- I don't want to lose my status. (SV03_09)
- I have concerns my dancing skills are going down. (SV03_10)
- I have concerns my body is getting out of its aesthetic form. (SV03_11)
- I want to impress the audience. (SV03_12)
- Dancing is more important to me than my health. (SV03_13)
- Dancing is my passion – I just have to keep dancing. (SV03_14)

1.1 Total sample (n = 72)

	SV03_01	SV03_02	SV03_03	SV03_04	SV03_05	SV03_06	SV03_07
SV03_01	1						
SV03_02	.13112168	1					
SV03_03	.00310321	.02310316	1				
SV03_04	-.30826671	-.04740609	.43225761	1			
SV03_05	-.15405249	-.1942711	.39479968	.28694763	1		
SV03_06	-.22016889	-.03851218	.33141141	.61248138	.68337936	1	
SV03_07	-.20249914	-.00394235	.68810838	.54379154	.62553467	.59297379	1
SV03_08	-.18334899	-.0361152	.61391724	.43100055	.45610832	.41472189	.84102546
SV03_09	-.23808368	.13222995	.52245623	.64348634	.26430089	.61430805	.71169758
SV03_10	-.10298589	.05155111	.21977938	.57950127	.16510934	.54707232	.51590046
SV03_11	-.10514254	.11989243	.40428552	.60224817	.3610688	.63754526	.62135444
SV03_12	-.1817817	.09125127	.3934237	.48416535	.23887322	.38696451	.53733432
SV03_13	-.21194019	.26950167	.02767623	.45751447	.12397055	.43974823	.3095098
SV03_14	-.13908963	.26598537	.04530236	.34777886	.04775272	.11763157	.34469205

	SV03_08	SV03_09	SV03_10	SV03_11	SV03_12	SV03_13	SV03_14
SV03_08	1						
SV03_09	.77007775	1					
SV03_10	.54810185	.72319564	1				
SV03_11	.53856502	.78024982	.7147965	1			
SV03_12	.61656887	.81690247	.64600315	.72127575	1		
SV03_13	.33101017	.53259502	.64993565	.55588548	.70862288	1	
SV03_14	.3490483	.49806553	.68776847	.36463819	.52232506	.71176575	1

1.2 English version (n = 36)

	SV03_01	SV03_02	SV03_03	SV03_04	SV03_05	SV03_06	SV03_07
SV03_01	1						
SV03_02	.24844975	1					
SV03_03	-.16698161	.20051907	1				
SV03_04	-.03580701	-.07537903	.66307471	1			
SV03_05	-.39171336	-.24997922	.6149999	.75207871	1		
SV03_06	-.40147483	-.26854052	.6191515	.8797994	.80270811	1	
SV03_07	-.33587097	-.07660662	.67318993	.67879471	.70202147	.59885553	1
SV03_08	-.52499331	-.03434543	.55018912	.56882634	.5604853	.62253231	**.90555383**
SV03_09	-.26475914	-.1917165	.65039753	.66368793	.60934775	.70831185	.84807984
SV03_10	-.24866486	-.10416417	.33964099	.56073622	.26747853	.60621665	.74253774

SV03_11	.06121312	-.1146902	.38929674	.70035157	.5587086	.65222396	.48285312
SV03_12	-.27439922	-.45612597	.37681066	.69188583	.47835021	.62908109	.68836115
SV03_13	-.29186847	-.17193946	.19861623	.51472752	.32237813	.44570606	.50945299
SV03_14	-.37938644	.12810369	.46468068	.52552179	.38281989	.23595272	.76911409

	SV03_08	SV03_09	SV03_10	SV03_11	SV03_12	SV03_13	SV03_14
SV03_08	1						
SV03_09	.88345658	1					
SV03_10	.7715417	.73234967	1				
SV03_11	.47410473	.68703644	.6532235	1			
SV03_12	.75180778	**.91594562**	.75463043	.62800504	1		
SV03_13	.68655134	.6681693	.77970642	.62997268	.70865776	1	
SV03_14	.74291639	.6570142	.7185664	.3776977	.62300191	.77573269	1

1.3 German version (n = 36)

	SV03_01	SV03_02	SV03_03	SV03_04	SV03_05	SV03_06	SV03_07
SV03_01	1						
SV03_02	.03642206	1					
SV03_03	.21102857	-.11608962	1				
SV03_04	-.35689223	-.04822786	.41329616	1			
SV03_05	.02456155	-.14500111	.18389566	.0906335	1		
SV03_06	-.01459121	.13580954	.13605979	.43237583	.63126233	1	
SV03_07	-.01759447	.06755364	.6975963	.52680398	.56502373	.59489006	1
SV03_08	.2325762	-.0221743	.69273737	.37413076	.36884271	.23967562	.777116
SV03_09	-.08815855	.37717948	.49273638	.50887481	.05735145	.54480776	.63283467
SV03_10	.25194802	.19723294	.15325839	.47879469	.13755429	.49416573	.28606649
SV03_11	-.18067258	.31454534	.41239663	.54361961	.24079395	.62678774	.71833127
SV03_12	-.03254937	.50542063	.40956545	.2986338	.04659192	.16755829	.4085689
SV03_13	-.03235107	.67917413	-.11330045	.34499344	-.01440236	.41634116	.09246953
SV03_14	.19415941	.43454698	-.29239239	.12168535	-.28607058	-.02046691	-.13258639

	SV03_08	SV03_09	SV03_10	SV03_11	SV03_12	SV03_13	SV03_14
SV03_08	1						
SV03_09	.73700831	1					
SV03_10	.31685127	.65409166	1				
SV03_11	.59736718	.85264633	.76520749	1			
SV03_12	.52027566	.72779727	.51208265	.78182214	1		
SV03_13	-.06025468	.40545851	.44733118	.50767973	.74795516	1	
SV03_14	-.09969449	.33295766	.60443783	.31022773	.41873678	.62264303	1
