# Supplementary material for: Development of a Multidimensional Pain Questionnaire in Professional Dance (MPQDA): a pilot study
Source: BMC Sports Sci Med Rehabil. 2022 Nov 3;14:189. doi: 10.1186/s13102-022-00580-5 (PMC9635190; doi:10.1186/s13102-022-00580-5)
Supplement: Supplementary file 3 — Additional file 3. Multidimensional Pain Questionnaire in Professional Dance (MPQDA). [English version]. [file 13102_2022_580_MOESM3_ESM.pdf]

# Multidimensional Pain Questionnaire in Professional Dance (MPQDA)

This questionnaire is addressed to professional dancers with pain in the last 3 months which occurred during or within 24 hours after work time (in training, rehearsals and/or performances). Information on scoring can be found at the bottom (p.7).

## A. Sociodemographic and health related information

1. Which gender do you have?
   ☐ female
   ☐ male
   ☐ other/ diverse

2. How old are you?
   _________ years

3. What is your height?
   ________ centimetres (cm)

4. What is your weight?
   _______ kilograms (kg)

5. Do you currently have any injuries? (e.g. muscle strain in the upper thigh)
   ☐ no
   ☐ yes, following:___________________________________________________

6. Do you have any diseases? (e.g. high blood pressure, iron deficiency, scoliosis)
   ☐ no
   ☐ yes, following:___________________________________________________

7. Do you smoke?
   ☐ no
   ☐ yes, up to 10 cigarettes a day
   ☐ yes, more than 10 cigarettes a day

## B. Information on professional practice

8. How many years have you been working as a dancer?
   (Number of years in which you earn your livelihood by dancing.)
   ___________ years

9. What is your current employment relationship as a dancer?
   ☐ freelance
   ☐ salaried as
      ☐ group dancer (Corps de ballet)
      ☐ group dancer (Corps de ballet) with solo contract
      ☐ soloist

10. Which dance genre do you practice predominantly during the week?
    ☐ Classical Dance (Ballet)/ Neoclassical Dance
    ☐ Contemporary Dance/ Dance Theatre
    ☐ Musical/ Revue
    ☐ Other:____________________________

11. How many hours do you spend on average per week with dance training, rehearsals and performances?

| | | |
|---|---|---|
| dance training: | ________ | hours per week |
| rehearsals: | ________ | hours per week |
| performances: | ________ | hours per week |

## C. Pain questions

12. Have you had any pain in the last 3 months which occurred during or within 24 hours after work time (training/ rehearsals/ performances)?
    ☐ no
    ☐ yes

*All the following questions relate to the* ***pain within the last 3 months****. Please answer the following questions if you have answered "yes" in the previous question.*

13. Please indicate the painful body region(s) within the last 3 months which occurred during or within 24 hours after work time (training/ rehearsals/ performances).
    (Multiple answers are possible)

| **Head and torso** | |
|---|---|
| head | ☐ |
| neck/ cervical spine | ☐ |
| upper back/ thoracic spine | ☐ |
| lower back/ lumbar spine/ iliosacral joint | ☐ |
| stomach | ☐ |

| **Upper extremity** | **right** | **left** |
|---|---|---|
| shoulder/ upper arm | ☐ | ☐ |
| elbow/ forearm | ☐ | ☐ |
| wrist/ hand | ☐ | ☐ |

| **Lower extremity** | **right** | **left** |
|---|---|---|
| hip joint | ☐ | ☐ |
| upper thigh | ☐ | ☐ |
| knee | ☐ | ☐ |
| lower leg | ☐ | ☐ |
| ankle joint | ☐ | ☐ |
| rear-/ midfoot | ☐ | ☐ |
| forefoot | ☐ | ☐ |

14. Please indicate the <u>most severely</u> affected pain region within the last 3 months which occurred during or within 24 hours after work time (training/ rehearsals/ performances).
Please indicate the region as precisely as possible. If pain is more generalized, you can select one of the main categories (e.g. upper extremity). If you only specified one region in the previous question, enter it again.

| | |
|---|---|
| **Head and torso** | ☐ |
| head | ☐ |
| neck/ cervical spine | ☐ |
| upper back/ thoracic spine | ☐ |
| lower back/ lumbar spine/ iliosacral joint | ☐ |
| stomach | ☐ |

| **Upper extremity** | ☐ | |
|---|---|---|
| | **right** | **left** |
| shoulder/ upper arm | ☐ | ☐ |
| elbow/ forearm | ☐ | ☐ |
| wrist/ hand | ☐ | ☐ |

| **Lower extremity** | ☐ | |
|---|---|---|
| | **right** | **left** |
| hip joint | ☐ | ☐ |
| upper thigh | ☐ | ☐ |
| knee | ☐ | ☐ |
| lower leg | ☐ | ☐ |
| ankle joint | ☐ | ☐ |
| rear-/ midfoot | ☐ | ☐ |
| forefoot | ☐ | ☐ |

*All of the following questions refer to the* ***most severely affected pain region*** *from the previous question.*

15. The pain region is ...

| | **not** | **somewhat** | **fairly** | **very** |
|---|---|---|---|---|
| tight/ hard/ tense | ☐ | ☐ | ☐ | ☐ |
| restricted in mobility | ☐ | ☐ | ☐ | ☐ |
| less resilient | ☐ | ☐ | ☐ | ☐ |

16. Please indicate your average perceived pain intensity within the last 3 months between no pain (= 0) and worst conceivable pain (= 10).

no pain — worst conceivable pain

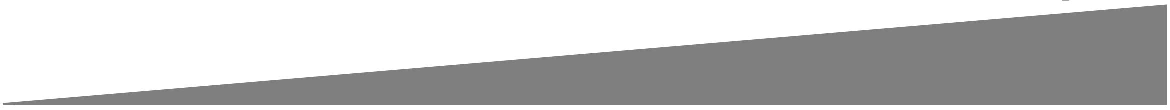

| 0 | 1 | 2 | 3 | 4 | 5 | 6 | 7 | 8 | 9 | 10 |
|---|---|---|---|---|---|---|---|---|---|---|
| ☐ | ☐ | ☐ | ☐ | ☐ | ☐ | ☐ | ☐ | ☐ | ☐ | ☐ |

17. I feel the pain as....

| | **does not apply** | **applies a little** | **applies mostly** | **applies exactly** |
|---|---|---|---|---|
| pulling | ☐ | ☐ | ☐ | ☐ |
| tearing | ☐ | ☐ | ☐ | ☐ |
| shooting | ☐ | ☐ | ☐ | ☐ |
| stabbing | ☐ | ☐ | ☐ | ☐ |
| sharp | ☐ | ☐ | ☐ | ☐ |
| fearful | ☐ | ☐ | ☐ | ☐ |
| wretched | ☐ | ☐ | ☐ | ☐ |
| terrible | ☐ | ☐ | ☐ | ☐ |
| paralyzing | ☐ | ☐ | ☐ | ☐ |
| unbearable | ☐ | ☐ | ☐ | ☐ |

18. Please indicate since when the pain occurred and how often it has occurred in the selected period (rarely to permanently).

☐ **maximum of one week**
☐ rarely ☐ frequently ☐ permanently

☐ **between one week and 6 weeks**
☐ rarely ☐ frequently ☐ permanently

☐ **between 6 and 12 weeks**
☐ rarely ☐ frequently ☐ permanently

☐ **between 3 and 6 months**
☐ rarely ☐ frequently ☐ permanently

☐ **longer than 6 months**
☐ rarely ☐ frequently ☐ permanently

19. The onset of pain occurred...
☐ suddenly/ through a single (traumatic) event
☐ over time/ creeping
☐ within 24 hours after work time (training/ rehearsals/ performances)
☐ in another way, as follows: ____________________________________________

20. The pain occurs...

| | **never** | **rarely** | **frequently** | **permanently** |
|---|---|---|---|---|
| when weight-bearing | ☐ | ☐ | ☐ | ☐ |
| during movement | ☐ | ☐ | ☐ | ☐ |
| at rest | ☐ | ☐ | ☐ | ☐ |

21. Please indicate to what extent you feel the pain to be good/positive (rather harmless, not disturbing) or bad/negative (alarming, disturbing) within the last 3 months.

good/positive pain — bad/negative pain

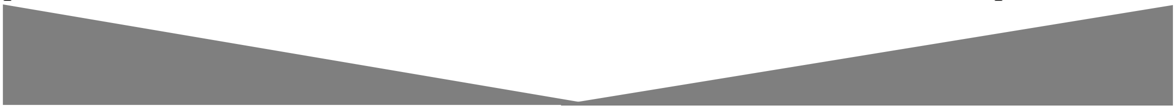

| +5 | +4 | +3 | +2 | +1 | 0 | -1 | -2 | -3 | -4 | -5 |
|---|---|---|---|---|---|---|---|---|---|---|
| ☐ | ☐ | ☐ | ☐ | ☐ | ☐ | ☐ | ☐ | ☐ | ☐ | ☐ |

22. Do you work despite pain (in training/ rehearsals/ performances)?

| | **No** | **Yes, with limitations** | **Yes, without limitations** |
|---|---|---|---|
| in training | ☐ | ☐ | ☐ |
| in rehearsals | ☐ | ☐ | ☐ |
| in performances | ☐ | ☐ | ☐ |

*If you work despite pain, i.e. if you have indicated "yes" to at least one category in the previous question, also answer the following question.*

23. Why do you work despite pain?

| | **does not apply** | **applies a little** | **applies mostly** | **applies exactly** |
|---|---|---|---|---|
| I don't want to let my company down. | ☐ | ☐ | ☐ | ☐ |
| I feel existential/ financial pressure. | ☐ | ☐ | ☐ | ☐ |
| I feel pressure from superiors (training leader, choreographer). | ☐ | ☐ | ☐ | ☐ |
| I feel pressure from colleagues. | ☐ | ☐ | ☐ | ☐ |
| I don't want to be considered unreliable. | ☐ | ☐ | ☐ | ☐ |
| I don't want to lose my role. | ☐ | ☐ | ☐ | ☐ |
| I don't want to lose my status. | ☐ | ☐ | ☐ | ☐ |
| I have concerns my dancing skills are going down. | ☐ | ☐ | ☐ | ☐ |
| I have concerns my body is getting out of its aesthetic form. | ☐ | ☐ | ☐ | ☐ |
| I want to impress the audience. | ☐ | ☐ | ☐ | ☐ |
| Dancing is more important to me than my health. | ☐ | ☐ | ☐ | ☐ |
| Dancing is my passion – I just have to keep dancing. | ☐ | ☐ | ☐ | ☐ |

Other/ additional reasons:

________________________________________

________________________________________

## Scoring of pain questions (Block C)

| Question | Score | Formation of a sum score* |
|---|---|---|
| Q 12† | not applicable | not applicable |
| Q 13: Head and torso | per region selected = 1 point | yes (minimum sum = 0,<br>maximum sum = 5) |
| Q 13: Upper extremity | per region selected = 1 point | yes (minimum sum = 0,<br>maximum sum = 6) |
| Q 13: Lower extremity | per region selected = 1 point | yes (minimum sum = 0,<br>maximum sum = 14) |
| Q 14‡ | not applicable | not applicable |
| Q 15 | not = 0 points<br>somewhat = 1 point<br>fairly = 2 points<br>very = 3 points | no, separate scoring per item |
| Q 16 | equals the marked point value on the scale (0 to 10) | no, separate scoring |
| Q 17: Sensory quality („pulling“ to „sharp“) | does not apply = 0 points<br>applies a little = 1 point<br>applies mostly = 2 points<br>applies exactly = 3 points | yes (minimum sum = 0,<br>maximum sum = 15) |
| Q 17: Affective quality („fearful“ to „unbearable“) | does not apply = 0 points<br>applies a little = 1 point<br>applies mostly = 2 points<br>applies exactly = 3 points | yes (minimum sum = 0,<br>maximum sum = 15) |
| Q 18: Duration | maximum of 1 week = 0 points<br>1 week to 6 weeks = 1 point<br>6 to 12 weeks = 2 points<br>3 to 6 months = 3 points<br>longer than 6 months = 4 points | no, separate scoring |
| Q 18: Frequency | rarely = 1 point<br>frequently = 2 points<br>permanently = 3 points | no, separate scoring |
| Q 19‡ | not applicable | not applicable |
| Q 20 | never = 0 points<br>rarely = 1 point<br>frequently = 2 points<br>permanently = 3 points | no, separate scoring per item |
| Q 21 | +5 = 0 points, +4 = 1 point,<br>+3 = 2 points, +2 = 3 points,<br>+1 = 4 points, 0 = 5 points,<br>-1 = 6 points, -2 = 7 points,<br>-3 = 8 points, -4 = 9 points,<br>-5 = 10 points | no, separate scoring |
| Q 22 | No = 2 points<br>Yes, with limitations = 1 point<br>Yes, without limitations = 0 points | no, separate scoring per item |
| Q 23 | does not apply = 0 points<br>applies a little = 1 point<br>applies mostly = 2 points<br>applies exactly = 3 points | yes (minimum sum = 0,<br>maximum sum = 36) |

†This pain questionnaire is for dancers who answer "yes" to question 12. The following questions are validated for pain in the last 3 months.

‡The questions are important in content, but cannot be scored numerically because the response options are equal-ranking categories.

*The point values of the individual items can be summed up.
