# Supplementary material for: Development of a Multidimensional Pain Questionnaire in Professional Dance (MPQDA): a pilot study
Source: BMC Sports Sci Med Rehabil. 2022 Nov 3;14:189. doi: 10.1186/s13102-022-00580-5 (PMC9635190; doi:10.1186/s13102-022-00580-5)
Supplement: Supplementary file 4 — Additional file 4. Multidimensionaler Schmerzfragebogen im professionellen Tanz (MPQDA). [German version]. [file 13102_2022_580_MOESM4_ESM.pdf]

Multidimensionaler Schmerzfragebogen im professionellen Tanz (MPQDA)

Dieser Fragebogen richtet sich an professionelle Tänzer*innen mit Schmerzen innerhalb der letzten 3 Monate während der Arbeitszeit (in Training, Proben und/ oder Vorstellungen) oder innerhalb von 24 Stunden danach. Informationen zur Auswertung befinden sich am Ende (S.7).

A. Soziodemographische und gesundheitsbezogene Angaben

1. Welches Geschlecht haben Sie?
   ☐ weiblich
   ☐ männlich
   ☐ anders/divers

2. Wie alt sind Sie?
   _________ Jahre

3. Wie groß sind Sie?
   ________ Zentimeter (cm)

4. Wieviel wiegen Sie?
   _______ Kilogramm (kg)

5. Liegen bei Ihnen aktuell Verletzungen vor? (z.B. Muskelzerrung im Oberschenkel)
   ☐ nein
   ☐ ja, folgende:____

6. Liegen bei Ihnen Erkrankungen vor? (z.B. hoher Blutdruck, Eisenmangel, Skoliose)
   ☐ nein
   ☐ ja, folgende:____

7. Rauchen Sie?
   ☐ nein
   ☐ ja, bis zu 10 Zigaretten am Tag
   ☐ ja, mehr als 10 Zigaretten am Tag

B. Angaben zur Berufsausübung

8. Seit wie vielen Jahren sind Sie als Tänzer*in berufstätig?
   (Anzahl der Jahre, in denen Sie Ihren Lebensunterhalt durch das Tanzen verdienen.)
   seit ___________ Jahren

9. In welchem Beschäftigungsverhältnis stehen Sie zurzeit als Tänzer*in?
   ☐ freiberuflich
   ☐ angestellt als
      ☐ Gruppentänzer*in (Corps de ballet)
      ☐ Gruppentänzer*in (Corps de ballet) mit Solovertrag
      ☐ Solist*in

10. Welche Tanzrichtung üben Sie überwiegend in der Woche aus?
    ☐ Klassischer Tanz (Ballett)/ Neoklassischer Tanz
    ☐ Zeitgenössischer Tanz (Contemporary Dance)/ Tanztheater
    ☐ Musical/ Revue
    ☐ Sonstige:___________________________

11. Wie viele Stunden verbringen Sie durchschnittlich in der Woche mit Tanztraining, Proben und Vorstellungen?
    Tanztraining: ________ Stunden pro Woche
    Proben: ________ Stunden pro Woche
    Vorstellungen: ________ Stunden pro Woche

C. Schmerzfragen

12. Hatten Sie innerhalb der letzten 3 Monate Schmerzen während bzw. innerhalb von 24 Stunden nach der Arbeitszeit (Training/ Proben/ Vorstellungen)?
    ☐ nein
    ☐ ja

*Alle folgenden Fragen beziehen sich auf die **Schmerzen innerhalb der letzten 3 Monate.** Beantworten Sie die nachfolgenden Fragen, wenn Sie in der vorangegangen Frage „ja" angegeben haben.*

13. Bitte geben Sie die schmerzhafte/n Stelle/n der letzten 3 Monate an, die während bzw. innerhalb von 24 Stunden nach der Arbeitszeit (Training/ Proben/Vorstellungen) aufgetreten sind. (Mehrfachantworten sind möglich)

**Kopf und Rumpf**	
Kopf	☐
Nacken/ Halswirbelsäule	☐
Oberer Rücken/ Brustwirbelsäule	☐
Unterer Rücken/ Lendenwirbelsäule/ ISG	☐
Bauch	☐

**Obere Extremität**	**rechts**	**links**
Schulter/ Oberarm	☐	☐
Ellenbogen/ Unterarm	☐	☐
Handgelenk/ Hand	☐	☐

**Untere Extremität**	**rechts**	**links**
Hüftgelenk	☐	☐
Oberschenkel	☐	☐
Kniegelenk	☐	☐
Unterschenkel	☐	☐
Sprunggelenk	☐	☐
Rück-/ Mittelfuß	☐	☐
Vorfuß	☐	☐

14. Bitte geben Sie die <u>am stärksten</u> betroffene Schmerzstelle der letzten 3 Monate an, die während bzw. innerhalb von 24 Stunden nach der Arbeitszeit (Training/ Proben/Vorstellungen) aufgetreten ist.
Bitte geben Sie die Stelle so genau wie möglich an. Ist der Schmerz mehr generalisiert, kann auch eine Oberkategorienausgewählt werden (z.B. obere Extremität). Wenn Sie in der vorangegangenen Frage nur eine Stelle angegeben haben, geben Sie diese erneut an.)

**Kopf und Rumpf**	☐
Kopf	☐
Nacken/ Halswirbelsäule	☐
Oberer Rücken/ Brustwirbelsäule	☐
Unterer Rücken/ Lendenwirbelsäule/ ISG	☐
Bauch	☐

**Obere Extremität**	☐	
	**rechts**	**links**
Schulter/ Oberarm	☐	☐
Ellenbogen/ Unterarm	☐	☐
Handgelenk/ Hand	☐	☐

**Untere Extremität**	☐	
	**rechts**	**links**
Hüftgelenk	☐	☐
Oberschenkel	☐	☐
Kniegelenk	☐	☐
Unterschenkel	☐	☐
Sprunggelenk	☐	☐
Rück-/ Mittelfuß	☐	☐
Vorfuß	☐	☐

*Alle folgenden Fragen beziehen sich auf die **am stärksten betroffene Schmerzstelle** aus der vorangegangenen Frage.*

15. Die Schmerzstelle ist…

	**nicht**	**etwas**	**ziemlich**	**sehr**
fest/ hart/ verspannt/ angespannt	☐	☐	☐	☐
in der Beweglichkeit eingeschränkt	☐	☐	☐	☐
in der Belastbarkeit eingeschränkt	☐	☐	☐	☐

16. Bitte geben Sie Ihre durchschnittlich empfundene Schmerzintensität innerhalb der letzten 3 Monate zwischen kein Schmerz (= 0) und schlimmster denkbarer Schmerz (= 10) an.

kein Schmerz — schlimmster denkbarer Schmerz

0	1	2	3	4	5	6	7	8	9	10
☐	☐	☐	☐	☐	☐	☐	☐	☐	☐	☐

17. Ich empfinde die Schmerzen als...

	**Trifft nicht zu**	**Trifft ein wenig zu**	**Trifft weitgehend zu**	**Trifft genau zu**
ziehend	☐	☐	☐	☐
reißend	☐	☐	☐	☐
einschießend/ blitzartig	☐	☐	☐	☐
stechend	☐	☐	☐	☐
scharf	☐	☐	☐	☐
beängstigend/ bedrohlich	☐	☐	☐	☐
elend	☐	☐	☐	☐
furchtbar	☐	☐	☐	☐
lähmend	☐	☐	☐	☐
unerträglich	☐	☐	☐	☐

18. Bitte geben Sie an, seit wann die Schmerzen auftreten und anschließend wie häufig diese im gewählten Zeitraum auftreten (selten bis immer).

☐ **maximal eine Woche**
☐ selten ☐ häufig ☐ immer

☐ **zwischen einer Woche und 6 Wochen**
☐ selten ☐ häufig ☐ immer

☐ **zwischen 6 und 12 Wochen**
☐ selten ☐ häufig ☐ immer

☐ **zwischen 3 und 6 Monaten**
☐ selten ☐ häufig ☐ immer

☐ **länger als 6 Monate**
☐ selten ☐ häufig ☐ immer

19. Das Auftreten der Schmerzen erfolgte…
    ☐ plötzlich/ durch ein einzelnes (traumatisches) Ereignis
    ☐ über die Zeit/ schleichend
    ☐ innerhalb von 24 Stunden nach der Arbeitszeit (Training/Proben/Vorstellungen)
    ☐ anders, folgendermaßen: ____

20. Die Schmerzen treten auf bei…

	**nie**	**selten**	**häufig**	**immer**
Belastung (z.B. mit Körpergewicht)	☐	☐	☐	☐
Bewegung	☐	☐	☐	☐
In Ruhe	☐	☐	☐	☐

21. Bitte geben Sie an, inwiefern Sie die Schmerzen innerhalb der letzten 3 Monate_als gut/positiv (eher unbedenklich, nicht störend) oder schlecht/negativ (bedenklich, störend) empfinden.

guter/positiver Schmerz | schlechter/negativer Schmerz

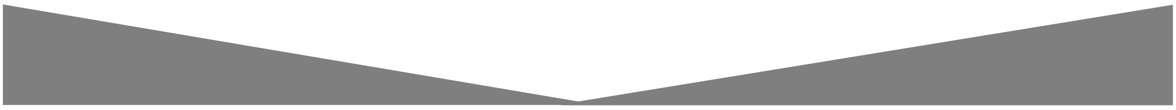

+5	+4	+3	+2	+1	0	-1	-2	-3	-4	-5
☐	☐	☐	☐	☐	☐	☐	☐	☐	☐	☐

22. Arbeiten Sie trotz der Schmerzen (in Training/Proben/Vorstellungen)?

	**Nein**	**Ja, mit Einschränkungen**	**Ja, ohne Einschränkungen**
im Training	☐	☐	☐
in Proben	☐	☐	☐
in Vorstellungen	☐	☐	☐

Wenn Sie trotz der Schmerzen arbeiten, d.h. wenn Sie in der vorangegangen Frage mindestens eine Kategorie mit „ja“ angegeben haben, beantworten Sie auch die nachfolgende Frage.

23. Warum arbeiten Sie trotz Schmerzen?

	**Trifft nicht zu**	**Trifft ein wenig zu**	**Trifft weitgehend zu**	**Trifft genau zu**
Ich will meine Company nicht im Stich lassen.	☐	☐	☐	☐
Ich empfinde existenziellen/ finanziellen Druck.	☐	☐	☐	☐
Ich empfinde Druck durch Vorgesetzte (Trainingsleiter*in, Choreograph*in)	☐	☐	☐	☐
Ich empfinde Druck durch Kolleg*innen.	☐	☐	☐	☐
Ich möchte nicht als unzuverlässig gelten.	☐	☐	☐	☐
Ich will meine Rolle nicht verlieren.	☐	☐	☐	☐
Ich will meinen Status nicht verlieren.	☐	☐	☐	☐
Ich habe Bedenken, dass meine tänzerischen Leistungen nachlassen.	☐	☐	☐	☐
Ich habe Bedenken, dass mein Körper aus der ästhetischen Form gerät.	☐	☐	☐	☐
Ich will das Publikum beeindrucken.	☐	☐	☐	☐
Tanzen ist mir wichtiger als meine Gesundheit.	☐	☐	☐	☐
Tanzen ist meine große Leidenschaft – ich muss einfach weiter tanzen.	☐	☐	☐	☐

Andere/ weitere Gründe, folgende:

____

____

Auswertung der Schmerzfragen (Block C)

**Frage**	**Punkte**	**Bildung eines Summenscore***
F 12†	entfällt	entfällt
F 13: Kopf und Rumpf	pro ausgewählte Region = 1 Punkt	Ja (minimale Summe = 0, maximale Summe = 5)
F 13: Obere Extremität	pro ausgewählte Region = 1 Punkt	Ja (minimale Summe = 0, maximale Summe = 6)
F 13: Untere Extremität	pro ausgewählte Region = 1 Punkt	Ja (minimale Summe = 0, maximale Summe = 14)
F 14‡	entfällt	entfällt
F 15	nicht = 0 Punkte<br>etwas = 1 Punkt<br>ziemlich = 2 Punkte<br>sehr = 3 Punkte	Nein, Einzelwertung pro Item
F 16	entspricht dem angekreuzten Punktwert auf der Skala (0 bis 10)	Nein, Einzelwertung
F 17: Sensorische Qualität („ziehend“ bis „scharf“)	trifft nicht zu = 0 Punkte<br>trifft ein wenig zu = 1 Punkt<br>trifft weitgehend zu = 2 Punkte<br>trifft genau zu = 3 Punkte	Ja (minimale Summe = 0, maximale Summe = 15)
F 17: Affektive Qualität („beängstigend/ bedrohlich“ bis „unerträglich“)	trifft nicht zu = 0 Punkte<br>trifft ein wenig zu = 1 Punkt<br>trifft weitgehend zu = 2 Punkte<br>trifft genau zu = 3 Punkte	Ja (minimale Summe = 0, maximale Summe = 15)
F 18: Dauer	maximal 1 Woche = 0 Punkte<br>1 Woche bis 6 Wochen = 1 Punkt<br>6 bis 12 Wochen = 2 Punkte<br>3 bis 6 Monaten = 3 Punkte<br>länger als 6 Monate = 4 Punkte	Nein, Einzelwertung
F 18: Frequenz	selten = 1 Punkt<br>häufig = 2 Punkte<br>immer = 3 Punkte	Nein, Einzelwertung
F 19‡	entfällt	entfällt
F 20	nie = 0 Punkte<br>selten = 1 Punkt<br>häufig = 2 Punkte<br>immer = 3 Punkte	Nein, Einzelwertung pro Item
F 21	+5 = 0 Punkte, +4 = 1 Punkt,<br>+3 = 2 Punkte, +2 = 3 Punkte,<br>+1 = 4 Punkte, 0 = 5 Punkte,<br>-1 = 6 Punkte, -2 = 7 Punkte,<br>-3 = 8 Punkte, -4 = 9 Punkte,<br>-5 = 10 Punkte	Nein, Einzelwertung
F 22	Nein = 2 Punkte<br>Ja, mit Einschränkungen = 1 Punkt<br>Ja, ohne Einschränkungen = 0 Punkte	Nein, Einzelwertung pro Item
F 23	trifft nicht zu = 0 Punkte<br>trifft ein wenig zu = 1 Punkt<br>trifft weitgehend zu = 2 Punkte<br>trifft genau zu = 3 Punkte	Ja (minimale Summe = 0, maximale Summe = 36)

†Dieser Schmerzfragebogen richtet sich an Tänzer*innen, die auf Frage 12 mit „ja“ antworten. Die folgenden Fragen sind für Schmerzen der letzten 3 Monate validiert.

‡Die Fragen sind inhaltlich wichtig, können aber nicht nummerisch gewertet werden, da es sich bei den Antwortmöglichkeiten um gleichrangige Kategorien handelt.

*Die Punktwerte der einzelnen Items können summiert werden.
